# Exploring the willingness toward HIV immediate test and treat among MSM in Nairobi and its environs: a cross-sectional study

**DOI:** 10.3389/fpubh.2023.1228709

**Published:** 2024-01-03

**Authors:** Kingori Ndungu, Peter Gichangi, Marleen Temmerman

**Affiliations:** ^1^Department of Public Health and Primary Care, Faculty of Medicine and Health Sciences, Ghent University, Ghent, Belgium; ^2^Department of Environment and Health Sciences, Technical University of Mombasa, Mombasa, Kenya; ^3^International Centre for Reproductive Health, Mombasa, Kenya; ^4^Aga Khan University Hospital, Nairobi, Kenya

**Keywords:** ART, HIV, HIVST, immedate test and treat, MSM, MSW

## Abstract

**Background:**

In the test and treat initiative, high-risk populations are screened for human immunodeficiency virus (HIV) infection and start early treatment if diagnosed positive. This study explores factors associated with willingness to initiate testing and immediate treatment among men who have sex with men (MSM) in Nairobi and its environs. The study was informed by a conceptual framework combining the AIDS Risk Reduction Model (ARRM) and the Modified Social Ecological Model.

**Methods:**

This cross-sectional exploratory study targeted MSM (aged 18–60 years) reporting active engagement in anal or oral sex with men in Nairobi and its surrounding areas. Purposive sampling was used to identify data collection sites, and then snowballing was employed to reach the respondents. Data analysis was performed using SPSS version 23, and binary logistics regression was used for inferential analysis.

**Results:**

Between July 2018 and June 2019, 391 MSM were recruited to fill out a self-administered questionnaire, out of which 345 complete questionnaires were analyzed. Never been tested for HIV, private/NGO as the facility of the last HIV test, and had unprotected anal sex were listed as the reasons for taking the most recent test, and the results of the most recent HIV test and seeking a post self-test confirmation were associated with a higher likelihood of accepting the immediate HIV test and treat initiative. Additionally, a preference for a health provider as the first source of support, belief in the efficacy of ARVs, and disclosure about being on ART were the other reasons. Additionally, being aged 25+ years, having more than 60 USD monthly income, and having inconsistent condom use during sex were associated with a higher likelihood of accepting the immediate HIV test and treat initiative. Barriers to the immediate test and treat strategy included stigma from healthcare providers and concerns about disruptions in lifestyle through antiretroviral therapy (ART) use.

**Conclusion:**

Interventions aimed at increasing the HIV test and treat initiative in Kenya may need to take into account the demographic and social characteristics of MSM, including age, lack of habitual HIV testing, and lifestyle changes before and upon enrollment in ART. Projects should also consider working closely with healthcare facilities to strengthen treatment preparation, especially for asymptomatic MSM and those who may not be immediately willing to choose the test and treat strategy.

## Background

Kenya had ~1.4 million adults living with the human immunodeficiency virus (HIV), and the HIV prevalence among men who have sex with men (MSM) was 18.2% in 2019 (1). One-third (33%) of all new HIV infections in Kenya occur among key populations, and HIV prevalence among MSM in Kenya is almost three times higher than the general population (2, 3). A steep reduction in incidences is required to halt the HIV epidemic in Kenya. UNAIDS aims to achieve 95–95–95 at a global level by 2030, and apart from Botswana (4), most of the other sub-Saharan African (SSA) nations are not on course to meet this UNAIDS target (5).

Early initiation of antiretroviral therapy (ART) significantly reduces HIV transmissions among couples (6, 7). Additionally, when HIV viral load is suppressed, HIV risk for transmission through anal sex is reduced significantly to zero (8–10). Test and treat is an intervention strategy aimed at eliminating HIV by reducing the spread of the virus to other people through screening at-risk populations for HIV and early treatment for the seropositive (11). The test and treat strategy offers two types of impact at the population level, namely, reduced opportunity for secondary transmission and (11) reduced infectiousness per contact. Mathematical modeling predicts that HIV incidences would significantly decline if the immediate test and treat option is provided throughout the population, even though HIV prevalence may not decrease and may increase (12).

HIV testing remains suboptimally implemented, especially within the MSM community. Many non-suppressed HIV-positive MSMs are asymptomatic with a high cluster of differentiation 4 (CD 4 cell) counts; such individuals may prove a challenge to test, engage in care, and virally suppress (13–16). However, the current challenge is finding HIV-infected individuals who are not receiving care and treatment and tailoring approaches that will ensure retention. A major challenge confronting HIV care and treatment programs in low- and middle-income countries is the late initiation of antiretroviral therapy (ART), challenges in retention, and high patient attrition sometimes due to stigma (17), and this situation may be even more dire among MSM. In addition, MSM in Africa has low engagement with the HIV treatment cascade despite improvements in HIV testing (18). The World Health Organization (WHO) released guidelines in 2015 for the immediate initiation of treatment for everyone diagnosed with HIV (19), which were adopted by Kenya in the same year (20). Additionally, the Ministry of Health (MOH) Kenya, in July 2016, released revised HIV prevention and treatment guidelines that called for immediate test and treat strategy. The new guidelines were received with mixed reactions, with many questions among the implementers about the feasibility of the strategy. Given these guidelines and the reactions to them, this study seeks to explore the HIV risk factors and other factors associated with willingness to immediately test and treat for HIV among the MSM in Nairobi, Kenya.

## Methods

### Study design and population

The conceptual framework combining the AIDS Risk Reduction Model (ARRM) and the Modified Social Ecological Model informed this study. A cross-sectional study design was used. All consenting adult men (18–60 years) who reported having engaged in consensual oral or anal sex with other men and living in Nairobi city and its neighboring Kiambu County were eligible to be part of the study population. The age limit of 18 years was chosen to reduce the ethical challenges of getting parental/guardian consent from minors. Identifying and recruiting the MSM is a doughty task due to the criminalization and stigmatization associated with homosexuality. Purposive sampling was used to identify 11 data collection points where the MSM community regularly meets because of the secretive nature of MSM activities as well as the criminalization of the same.

### Sampling techniques

The study reached 391 MSM who responded to the self-administered questionnaires, of which 345 had complete information. Places and venues frequented by the MSM community, such as safe spaces/drop-in centers, hotels, and massage parlors, were identified through purposive sampling. Other studies have suggested that in a given geographical environment, the same population is likely to be captured in different spaces within that environment. The snowball/chain referral method was used to reach respondents, as key/hidden populations typically have the same/similar set of networks and physical spaces they feel safe in and frequent. This method, despite its inherent flaws, was the most optimal to use to identify and reach this hidden population. Missing data were handled through a listwise approach that omitted those cases with the missing data and analyzed the remaining data. Listwise is a default option for analysis in most statistical software packages, including the Statistical Package for the Social Sciences (SPSS), which we used for the analysis (21).

### Data collection

Data collection tools were developed by consulting experts and the MSM and pretested with 45 respondents for comprehension, relevance, and completeness. Data collection for the study took place in the months of July 2018 to June 2019 after obtaining informed consent from the interviewees. English paper-based self-administered structured questionnaires were distributed. The questionnaires were structured in three sections: demographics and socio-economic status of the interviewees formed the first section; followed by questions on HIV risk behaviors in the second section; and the final section had questions on willingness, uptake of immediate HIV test and treat option, and its associated factors.

### Measures

The dependent variable for the study was a willingness to start ART immediately. The following socio-demographics were collected: age, place of birth, religion, education level, marital status, employment status, and income. Beyond that, the following risk factors were investigated: identifying as MSM/MSW, number of sexual partners, preference for top or bottom sexual positions, condom use, condom use after alcohol/hard drugs, and type of lubricant. The following variables were of interest in the willingness and uptake of the immediate HIV test and treat initiative: never tested for HIV; type of facility for the last HIV test; reason for the most recent test; never heard of the window period; if HIV positive through HIV self-test, would you go for a confirmatory test; would you go for counseling after positive result; if positive test result, who would you want to approach first to seek support; have you ever experienced stigma from healthcare providers; do you think ARV would affect or disrupt your lifestyle; and if you were on ART, would you prefer to have a treatment “buddy.” All of the above were binary variables.

### Data analysis and management

The data analysis process began with numbering the questionnaires. The data cleaning and management was conducted on a Microsoft Excel 2016 spreadsheet, which was then exported to IBM SPSS 23. In the SPSS Version 23.0 software database, variables were labeled and analyses were conducted. Proportions and their 95% confidence interval were calculated. Data that had inconsistencies between different sections were excluded from the study. Cross-tabulation was performed. Bivariate analysis was performed. The prevalence odds ratio (POR) and adjusted POR were calculated. Multivariable analysis was performed, including variables statistically significant on bivariate analysis or those known to have an impact on the variable of interest. A *p*-value of < 0.05 was considered statistically significant.

### Ethical considerations

The study obtained ethical clearance from the Ghent University approval number (PA 2016/009) and the Ethics Review Committee for Mount Kenya University (approval number: MKU/ERC/0463). The researchers also tried to ensure full compliance with the Declaration of Helsinki as the study involved human respondents. The study obtained informed consent from the interviewees. All identifiers that would potentially help to identify the interviewee in the data were omitted, and unique identifiers were used during data processing. Data were only accessible to researchers doing data analysis in secure databases and computers. The research team underwent comprehensive training on research ethics before the commencement of the study.

## Results

### Respondent demographic and socio-economic information

A majority of the respondents (64%) were under 25 years old, single (86.6%), and unemployed (61.7%). While all respondents had attained at least secondary education, their education levels were generally high (at least secondary and above). In total, 16% of the participants indicated that their education had been impacted by their sexual identity. More than half of respondents (58.8%) identified themselves as MSMs who engage in sex work (MSW; Table 1).

**Table 1:** Respondent demographic and socio-economic information.

	**Variable**	**Frequency**	**Percent**
Respondent's age	18–24 years	210	60.9
	25+ years	135	39.1
Place of birth	Kenya	291	86.6
	Non-Kenya	45	13.4
Religion	Christian	244	71.8
	Non-Christian	96	28.2
Educational level	At least secondary education	204	59.8
	Tertiary education	137	40.2
Sexuality identity impacted education	Yes	52	16.0
	No	272	84.0
Currently employed	Yes	116	38.3
	No	187	61.7
Monthly income	Less than 60 USD	94	47.2
	60+ USD	105	52.8
Identity as an MSM or MSW	MSM	142	41.2
	MSW	203	58.8
Marital status	Single	291	86.6
	Married	45	13.4

MSW, men who have sex with men; MSW, male sex workers; USD, United States dollar.

### Demographic and HIV risk factors associated with willingness to start ART immediately

Study respondents aged 25+ years (75.6%) were more likely to consider immediate HIV testing and treatment at POR 1.06 (1.03, 1.42). Monthly income had statistical significance with a POR of 0.93 (1.09, 1.79). There were no differences in the proportion of Kenyans (77.3%) and non-Kenyans (73.33%) in regard to immediate HIV testing and treatment. There was no observed statistical difference between Christians (79.5%) and non-Christians (70.8%) in accepting treatment immediately. Respondents earning above 60 USD (79.0%) reported willingness to test and treat with a POR of 0.93 (1.09, 1.79); this indicates that >60 USD income negatively influences willingness to test and treat, implying reduced chances of respondents with monthly income above 60 USD willingness to test and treat by 0.07 times. While there were no statistical differences observed between self-identified MSM (76.06%) and MSW, a higher proportion of MSW who were using condoms inconsistently [95 (72.5%)] reported that they would be willing to immediately enter into ART, with a POR of 1.1 (1.25, 1.97), implying that they were 0.1 times more likely to willingly be initiated into ART (Table 2).

**Table 2:** Bivariate analysis of demographic and HIV risk factors associated with willingness to start ART immediately.

**Variable**		**Willing to start ART immediately**		**Total**	**Pearson chi-square**	**Prevalence odds ratio (95% confidence interval)**
		**No**	**Yes**			
Respondent's age	18–24 years	36 (17.1)	174 (82.9)	210 (60.9)	0.023	Ref
	25+ years	33 (24.4)	102 (75.6)	135 (39.1)		1.06 (1.03, 1.42)
Place of Birth	Kenya	66 (22.7)	225 (77.3)	291 (86.6)	0.45	Ref
	Non-Kenya	12 (26.7)	33 (73.3)	45 (13.4)		1.05 (0.87, 1.27)
Religion	Christian	50 (20.5)	194 (79.5)	244 (71.7)	0.25	Ref
	Non-Christian	28 (29.1)	68 (70.8)	96 (28.2)		1.12 (0.97, 1.30)
Educational level	At least secondary education	49 (24.0)	155 (75.9)	204 (59.8)	0.56	Ref
	Tertiary education	28 (20.4)	109 (79.5)	137 (40.1)		0.95 (0.85, 1.07)
Monthly income	Less than 60 USD	25 (26.6)	69 (73.4)	94 (47.2)	0.025	Ref
	60+ USD	22 (20.9)	83 (79.0)	105 (52.7)		0.93 (1.09, 1.79)
Identity as an MSM or MSW	MSM	34 (23.9)	108 (76.0)	142 (41.1)	0.21	Ref
	MSW	45 (22.1)	158 (77.8)	203 (58.8)		1.6 (0.19, 1.87)
Marital status	Single	66 (22.6)	225 (77.3)	291 (86.6)	0.052	Ref
	Married	12 (26.6)	33 (73.3)	45 (13.3)		1.05 (0.87, 1.27)
Sexual partners last 6 months	One	12 (17.3)	57 (82.6)	69 (20.6)	0.25	Ref
	More than one	62 (23.4)	203 (76.6)	265 (79.3)		1.08 (0.95, 1.22)
Prefer top or bottom	Top	6 (5.7)	98 (94.2)	104 (89.6)	0.13	Ref
	Bottom	6 (10.3)	52 (89.6)	58 (50)		1.01 (0.94, 1.08)
Condom used during sex	Consistently	36 (20.1)	143 (79.8)	179 (57.7)	0.06	Ref
	Inconsistently	36 (27.4)	95 (72.5)	131 (42.2)		1.1 (1.25, 1.97)
Do you use a condom during anal sex after alcohol/hard drug use	Consistently	30 (19.4)	124 (80.5)	154 (52.7)	0.77	Ref
	Inconsistently	35 (25.3)	103 (74.6)	138 (47.2)		1.08 (0.95, 1.22)
Type of lubricant in the last sexual act	KY Jelly	45 (23.6)	145 (76.3)	190 (64.8)	0.053	Ref
	None are water-based	20 (19.4)	83 (80.5)	103 (35.1)		0.95 (0.84, 1.07)

MSW, men who have sex with men; MSW, male sex workers; USD, United States dollar.

### Uptake of immediate test and treat and other associated factors

Previous experience with HIV testing had a statistically significant POR of 1.26 (1.07, 1.77), indicating that the respondents who had never tested were 1.26 times more likely to start ART immediately; frequency of HIV testing had a statistically significant POR of 1.38 (1.11, 1.89), indicating that the respondents who routinely tested were 1.38 times more likely to start ART immediately; and the respondents type of facility visited in the last HIV/AIDS test was significant at POR 1.39 (1.18, 1.96), implying that the type of facility in the last visit was 1.39 times more likely to influence immediate ART treatment. The study respondents' reason for the most recent test, had a significant POR of 1.57 (1.23, 2.09), implying that the participant's reason for the most recent test was 1.57 times more likely to influence immediate ART start. The research participants who had never heard of the window period had a significant POR of 1.53 (1.12, 1.84), demonstrating that awareness of the window period was 1.53 times more likely to influence immediate ART start. The willingness to confirm HIV positive self-test had a statistically significant POR of 1.31 (1.16, 1.66), suggesting that the willingness to confirm HIV positive self-test was 0.31 times more likely to influence the immediate test of ART. The respondents who went for a confirmatory test after undertaking an HIV self-test within a month had a significant POR of 1.18 (1.04, 1.39), indicating that their willingness to undertake confirmatory testing was 0.18 times more likely to influence an immediate ART start. Respondents' willingness to go for counseling after a positive result had a statistically significant POR of 1.68 (1.15, 1.89), implying that the participants' willingness to go for counseling after a positive result was 0.68 times more likely to influence immediate ART start. Participants' opinion, in case of a positive test result, as to whom they would prefer to approach to seek support, was a significant POR of 1.41 (1.29, 1.79), meaning that the person approached first by the participants in case of a positive test result would influence immediate ART start 1.79 times. The respondents' opinion on the effectiveness of ART in managing HIV/AIDS was a significant POR of 1.55 (1.42, 1.72), implying that the respondents' opinion on the effectiveness of ART in managing HIV/AIDS was 0.55 times more likely to influence immediate ART start. Participants' opinion on the effect of ART on their lifestyle was statistically significant at 1.39 (1.17, 1.85), indicating that the participants' opinion on the effect of ART on their lifestyle was 1.39 times more likely to influence their immediate ART start. Participants' preference to have treatment buddies had a statistically significant POR of 1.42 (1.25, 1.91), meaning that treatment buddy preference by the study participants would influence the immediate start of ART 1.42 times. The above binary regression test was conducted at a 95% confidence interval (Table 3).

**Table 3:** Bivariate analysis on uptake of immediate test and treat and other associated factors.

**Variable**		**Willing to start ART immediately**		**Total**	**Pearson chi-square**	**Prevalence odds ratio (95% confidence interval)**
		**No**	**Yes**			
Never tested for HIV/AIDS	No	4 (23.5)	13 (76.4)	17 (4.9)	0.014	Ref
	Yes	75 (22.8)	253 (77.1)	328 (95.0)		1.26 (1.07, 1.768)
If yes, how often do you test for HIV/AIDS	Every 3 months	54 (23.0)	180 (76.9)	234 (67.8)	0.09	Ref
	More than 3 months	25 (22.5)	86 (77.4)	111 (32.1)		1.38 (1.11, 1.89)
Type of facility of the last HIV/AIDS test	Public	16 (17.5)	75 (82.4)	91 (29.3)	0.023	Ref
	Private/NGO	52 (23.7)	167 (76.2)	219 (70.6)		1.39 (1.18, 1.96)
Reasons for the most recent test	Routine	38 (18.6)	166 (81.3)	204 (68.2)	0.015	Ref
	Had unprotected anal sex	25 (27.2)	67 (72.8)	70 (31.8.)		1.57 (1.23, 2.09)
Never heard of the window period	No	50 (28.2)	127 (71.7)	177 (51.3)	0.015	Ref
	Yes	29 (17.2)	139 (82.7)	168 (48.7)		1.53 (1.12, 1.84)
If HIV positive through an HIV self-test, would you go for a confirmatory test	No	29 (43.2)	38 (56.7)	67 (19.4)	< 0.0001	Ref
	Yes	50 (17.9)	228 (82.0)	278 (80.5)		1.31 (1.16, 1.66)
If yes, after how long would you go for a confirmatory test	One month or less	64 (20.5)	247 (79.4)	311 (92.0)	0.016	Ref
	Won't go	11 (40.7)	16 (59.2)	27 (7.9)		1.18 (1.04, 1.39)
Would go for counseling after a positive result	No	53 (29.2)	128 (70.7)	181 (52.4)	0.003	Ref
	Yes	26 (15.8)	138 (84.1)	164 (47.5)		1.68 (1.15, 1.89)
If a positive test result, who would you want to approach first to seek support	Healthcare provider	57 (22.8)	192 (77.1)	249 (85.0)	0.028	Ref
	Partner	7 (15.9)	37 (84.0)	44 (15.0)		1.41 (1.29, 1.79)
Never heard of immediate test and treat	No	23 (21.1)	86 (78.9)	109 (37.5)	0.804	Ref
	Yes	36 (19.8)	145 (80.1)	181 (62.4)		0.49 (0.29, 0.160)
Do you think ARVs are effective in managing the HIV virus	No	9 (32.1)	19 (67.8)	28 (9.8)	0.05	Ref
	Yes	48 (18.8)	207 (81.1)	255 (90.1)		1.55 (1.42, 1.72)
Do you think ART would affect or disrupt your lifestyle	No	36 (18.4)	159 (81.5)	195 (68.6)	0.015	Ref
	Yes	23 (25.8)	66 (74.1)	89 (31.3)		1.39 (1.17, 1.85)
If you were on ART, would you prefer to have a “buddy” treatment	No	14 (21.5)	51 (78.4)	65 (26.6)	0.016	Ref
	Yes	34 (18.9)	145 (81.0)	179 (73.3)		1.42 (1.25, 1.91)

ART, antiretroviral therapy; ARV, antiretroviral; AIDS, acquired immunodeficiency syndrome; NGO, non-governmental organizations; HIV, human immunodeficiency virus.

### Multivariate analysis on uptake of immediate test and treat and associated factors

The covariates that had a statistically significant influence on willingness to immediately test and treat at the 5% level of significance were: respondent's age; never tested for HIV/AIDS; if never tested for HIV/AIDS, how often do you test for HIV/AIDS; type of facility last tested; reason for most recent test; never heard of window period; willingness to undertake a confirmatory test within a month; if yes, after how long would you go for a confirmatory test; and if tested HIV positive through HIV self-test, willingness to undergo counseling after positive HIV result. If a positive test result, who would you want to approach first to seek support if diagnosed with HIV; ARVs are effective in managing HIV; ARV would affect or disrupt lifestyle; experience stigma from healthcare providers; and preference for having treatment “buddy” if on ART were significantly associated with willingness to immediately test and treat with *p*-values of < 0.05. The above data can be found on Table 4.

**Table 4:** Multivariate binary logistics regression analysis of covariates on attitudes toward uptake of immediate test and treat and predictors.

**95% CI for APOR**	***p*-value**	**APOR**	**95% CI for APOR**	
			**Lower**	**Upper**
Respondent's age	0.028	1.98	1.75	2.81
Never tested for HIV/AIDS	0.02	1.23	1.16	1.84
If never tested for HIV/AIDS how often do you test for HIV/AIDS	0.017	1.92	1.87	2.42
Type of facility last tested	0.04	1.81	1.23	2.84
Reasons for the most recent test	0.02	1.13	1.09	1.56
Never heard of window period	0.04	0.54	0.13	2.21
If HIV positive through an HIV self-test, would you go for a confirmatory test	0.01	1.22	1.04	1.67
If yes, after how long would you go for a confirmatory test	0.049	1.670	1.16	2.58
Would go for counseling after a positive result	0.03	1.63	1.47	1.87
If a positive test result, who would you want to approach first to seek support	0.013	1.24	1.11	1.84
Do you think ART is effective in managing the HIV virus	0.018	1.35	1.13	1.88
Do you think ART will affect or disrupt your lifestyle	0.02	1.16	1.09	1.27
If you were on ART, would you prefer to have a treatment “buddy”	0.01	1.22	1.04	2.17

APOR, adjusted prevalence odds ratio; CI, confidence interval.

## Discussion

The findings suggest that age is associated with willingness to test and treat; a higher proportion of the respondents who were willing to enter into ART immediately after a positive HIV test result were young and possibly single MSM aged 18–24 years. There were statistically significant associations between immediate test and treat and having never tested for HIV. Respondents who tested in private or NGO facilities appeared more willing to test and treat, suggesting that the type of facility for the last HIV test has an influence on willingness to test and treat. Additionally, other factors that may influence the immediate uptake of ART include the reason for the most recent test, the results of the most recent HIV test, and seeking a confirmatory test upon a positive HIV result through a self-test. Barriers to immediately taking up test and treat included concerns about disruptions in lifestyle through ART use and stigma from healthcare providers.

It is important to note that ~51% of all new HIV cases in Kenya occur among adolescents and young people (15–24 years) (2, 3). Given the population demographics in Kenya (22), the willingness of young people (and specifically MSM) to enter into immediate ART is important in reducing the incidence of HIV in the coming generations. While there were no significant differences between MSM and MSW with respect to willingness to immediately enter into treatment, higher proportions of MSW than MSM were willing to enter into treatment, and this is critical as there is evidence that MSW is at a higher risk of infection than MSM (23–26).

The importance of immediate initiation into ART in reducing HIV incidence has been demonstrated by previous studies (27–32). Habitual HIV testing, awareness of test and treat, and willingness to protect self and others upon a positive test result, were also associated with the willingness to immediately enter into treatment, suggesting that knowledge of available options as well as personal attributes are potentially critical factors in the willingness to immediately test and treat.

Study findings also suggest that support systems are important in the quest for immediate treatment upon a positive HIV result, and this finding confirms those from other studies (33–38). Evidence also suggests that quality of care was associated with willingness to enter treatment upon a positive HIV result. There is evidence that positive provider attitudes are contributing to the willingness to immediately test and treat (39–44). Facilities that possibly had fewer judgmental providers (such as private and NGO facilities) were associated with more willingness to enter into ART than government/public facilities. The possibility of seeking support from providers, the availability of posttest counseling services, having a treatment buddy, and the ability to disclose to a partner that one was on ART were all associated with willingness to immediately enter into ART upon testing positive for HIV.

A number of factors would prevent the MSM from enrolling in care and treatment immediately after testing HIV positive, which include fear of ART affecting or disrupting lifestyle, side effects, and a poor service provider attitude toward stigma. Being tested, knowing their status, and starting ART treatment immediately posed a challenge to many participants, who felt that more time would be needed to prepare and be able to integrate the treatment regimen into their daily life schedules (32).

Our study presents crucial findings that would help better refine and implement the immediate test and treat initiative in Kenya, especially within the MSM community. This study had the following limitations. First, the respondents for this study were recruited from MSM-friendly projects. The projects are likely to provide sensitizations and training related to immediate testing and treating. Such selection bias can limit our generalizability. Second, due to the non-probability sampling techniques used during recruitment, MSM who participated in the study may not be representative of MSM in Nairobi and its surrounding areas.

## Conclusion

This study explored the facilitating and inhibiting factors concerning the immediate test and treat strategy among the MSM community in Nairobi, Kenya. Interventions aimed at increasing the acceptance of the immediate test and treat initiative in Kenya may need to take into account the demographic and social characteristics of MSM, which include age, lack of habitual HIV testing, addressing lifestyle changes before and upon enrollment in ART, and safe environments for MSM. Projects should also consider working closely with healthcare facilities to strengthen treatment preparation, especially for asymptomatic MSM, and ensure close follow-ups for MSM under immediate test and treat programs.

## Data availability statement

The original contributions presented in the study are included in the article/Supplementary material, further inquiries can be directed to the corresponding author/s.

## Ethics statement

The studies involving humans were approved by the study obtained ethical clearance from the Ghent University Approval number (PA 2016/009) and the Ethics Review Committee for Mount Kenya University (Approval number: MKU/ERC/0463). The studies were conducted in accordance with the local legislation and institutional requirements. The participants provided their written informed consent to participate in this study.

## Author contributions

KN was responsible for the formulation of the study, conducting data collection, analyzing, and developing the article. PG and MT reviewed the manuscript and provided technical advice. All authors read, approved the final manuscript, and made significant contributions to the conceptualization and design of this study.
