# Supplementary material for: Exploring the willingness toward HIV immediate test and treat among MSM in Nairobi and its environs: a cross-sectional study
Source: Front Public Health. 2024 Jan 3;11:1228709. doi: 10.3389/fpubh.2023.1228709 (PMC10792060; doi:10.3389/fpubh.2023.1228709)
Supplement: Supplementary file 1 [file Data_Sheet_1.pdf]

5.0 Annexures

5.1 Questionnaire

Exploring willingness towards HIV Immediate Test & Treat among the MSM in Nairobi and its environs. A cross sectional study.

Respondents Characteristics

I would like to ask you some general questions about yourself.

100. Kindly indicate the following details below.

Your names first letter	
Your mothers name first letter	
Date of birth	
District of Birth	

101. What is your Age ? please indicate.................

102. Place of Birth (Country)............................

103 Place of Residence ....

104 What is your Religion?

1□Roman Catholic
2□Protestant
3□Muslim
4□Traditional
5□Hindu
6□Buddhist
7□Others

105. Whats is your highest level of Educational Achievement ?

1□ Never
2□ Primary School
3□ Secondary School
4□ Tertially education

106.Has your sexual identity impacted on your educational attainment?

1□ Yes 2□ No

□ If yes, Kindly explain how.

....

107 Are you currently employed ?

1□ Yes 2 □ No

a) If yes, *tick appropriately*

1□ Full-time paid employment
2□ Part-time paid employment
3□ Self employed
4□ Un-employed

b) Whats your monthly income.
1 Less than 6,000
2 6,000-25,000
3 25,001-75,000
4 More than 75,000

108. Do you identify yourself as "men who have sex with men" (MSM)
1 Yes 2 No
a) If yes how long have you been an MSM.......................

109 Do you identify yourself as" men sex worker" (MSW)
1 Yes 2 No

110 Do you identify yourself as Transgender?
1 Yes 2 No

111.What is your sexual orientation
1 Homosexual
2 Bisexual
3 Heterosexual
4 others

If others, kindly specify............

112. Marital status
1 Single 4 Divorced /Seperated.
2 Windower
3 Married
If ever Married;
1 Were you married to a man
2 Were you married to a woman

113. Sexual attraction: How do you describe your feelings of sexual attraction at the moment?
1 As a man who is attracted to men
2 As a man who is attracted to a woman
3 Attracted to both sexes
4 Unsure.

114. How many sexual partners have you had sex with in the last six months?
1 One 2 Two 3 Multiple 4 None

115. Do you use condom during sex?
1 Yes 2 No

If, yes how often

1 Always 2 Most of the time 3 Sometimes 4 Never

116. Do you prefer top or bottom?
1 Top 2 Bottom 3 Both/Versatile

117. Are you currently using the following?

1 Alcohol 2 Hard drugs

If yes, to hard drugs, kindly, indicate which ones?.................................

118. Have you ever had Anal sex after Alcohol/Hard drugs use ?
1 Yes 2 No

119. Do you use condoms during anal sex after alcohol/Hard drugs use?
1 Always 2 Most of the time 3 Sometimes 4 Never

120. Did you use a lubricant in your last sexual act?
1 Yes 2 No

a) **If yes, Kindly tick where appropriate**
1 KY jelly
2 Body cream
3 Saliva
4 Vaseline

If others, kindly specify ……………..

121. Have you ever heard any mental health issues or disorder?
1. Yes 2. No

If yes, kindly explain…………….

122. Do you have an insurance cover?
1 Yes 2 No

123 Have you been tested for;

a) Testing

	Infection	Ever in life	In last 6 months	Knows status, **-ve**	Knows status, **+ve**
i.	HIV/AIDS	( )	( )	( )	( )
ii.	Tuberculosis	( )	( )	( )	( )
iii.	Hepatitis C	( )	( )	( )	( )
iv.	STI	( )	( )	( )	( )
v.	Hepatitis B	( )	( )	( )	( )

b) Are you currently enrolled in any HIV Prevention Care & Treatment program.
1 Yes 2 No

If Yes, kindly specify ………………..

124. What social media pages do you frequent most? ***Kindly select one***

1 Facebook 2 Whats-up

3 Instagram 4 Grinder

5 Gay.com 6 Badoo

7 Planet Romeo 8 Dating Buzz

9 Sex Trader

5.0 Uptake of Immediate Test & Treat

Definition of test & treat-HIV positive patient to start ART as soon as one is ready but within two weeks.

162 . Have you ever heard of immediate test & treat

1. Yes 2. No

163. How did you hear about immediate test & treat. (please tick)

1 Friend/Partner

2 Website / Research Publication

3 Social Media

4 Medical provider

5 Any other (specify)…………………………

164. Would you consider taking up ART therapy immediately as one of the HIV combination prevention strategy?

1 Yes 2 No

165. **If you were tested and found to be HIV infected would you start Anti-retroviral therapy (ART) immediately?**

1. Yes 2. No

a) If no why? (Probe)..

166. Would you recommend your partner to start medication immediately after testing positive

1. Yes 2. No

If no explain..

167. What do you think of taking ARVs immediately after testing positive and yet some one feels "healthy"....................

168. If you were on ARV would you still use protection (condom)

1. Yes 2. No

169. Do you think ARVs are effective in managing the HIV Virus

1 Yes 2. No

If No, kindly explain why....................................

170. What would prevent you from enrolling into care & treatment immediately after testing HIV positive?

1. Stigma

2. Lack of knoweldge

3 □ Fear

4 □ Service provider attitude

5.□ Not available

6 □ Others specify..............................

171. Do you think ARV would affect or disrupt your lifestyle

1□ Yes 2. □ No

a) If yes kindly explain how................................

172. Would you tell your partner you are taking anti-retrovirals (ARV)?

1. □ Yes 2. □ No

a) **What do you think would be their reaction?...........................**

173. How often is viral load test done

...

174. What do you think would prevent you from taking ARV consistently?

……………………………………………

175. What fears would you be having on starting ARVs immediately?

………………

176. Would you be knowing your viral load results?

1□ Yes 2. □ No

a) **If yes, what is the results?**

1□Suppressed 2□Un-suppresed

c). If on ARVS, on average how many days do you miss taking your pills?

…………………………….

d) If you miss what are the common reasons that you miss?

……………………………

e). Have you ever experinced stigma cause of taking ARVs

1□ Yes 2. □ No

If yes from whom?

…………………………….

General Questions

177. If you were ART would you prefer to have a treament "buddy"

1□ Yes 2. □ No

178 Do you have any additional questions or Information?

....

179. Do you know any other method of HIV prevention apart from the 4 above?

Kindly, indicate...........................

Thank you for your time
