# Supplementary material for: Exploring the willingness toward HIV immediate test and treat among MSM in Nairobi and its environs: a cross-sectional study
Source: Front Public Health. 2024 Jan 3;11:1228709. doi: 10.3389/fpubh.2023.1228709 (PMC10792060; doi:10.3389/fpubh.2023.1228709)
Supplement: Supplementary file 2 [file Data_Sheet_2.pdf]

Region	SN	First_Letter	Mother_Letter	DOB	District	Age	AgeREC	
	Serial Number	First Letter	Mother's name first Letter	Date of Birth	District of Birth	Age	Respondents age	
	151	J	H	24-Feb-00	Kiambu		18	0
	158	A	J	26-Jul-97	Kiambu		21	0
	159	B	J	22-Aug-99	Kiambu		19	0
	170	D	M	31-Dec-86	Kiambu		22	0
	171	A	E	15-Jun-93	Gatundu		25	1
	207	J						
	218	J	E	25-Jul-95	Waguthu		24	0
	222	Z	B				30	1
	242	M	S	15-Jun-94	Nairobi		24	0
	353	C	N		Kiambu		31	1
HOYMAS	1	R	J	11-Dec-93	Nairobi		23	0
	2	K	J	08-Jul-88	Nairobi		30	1
	5	K	A		Mombasa		26	1
	6	A	R		Nakuru		27	1
	8	K	J	16-Dec-86	Nyeri		31	1
	9	P	O	01-Jun-91	Nyando		27	1
	10	S	H	28-Jan-88	Kampala		30	1
	11	O	J	26-Nov-92	Siaya		26	1
	12	A	G	12-Aug-86	Kisoro		32	1
	14	F	R	20-Dec-97	Kaspul		21	0
	15	K	N	13-Feb-82	Nairobi		35	1
	17	S	J	03-Apr-96	Nairobi		22	0
	18	J	R	12-Dec-95	Nyeri		23	0
	19	M	N	15-Jun-94	Nairobi		24	0
	20	K	J	26-Feb-93	Kampala		24	0
	22	N	M	15-Mar-83	Masisi		35	1
	24	J	J	24-Oct-91	Kiambu		27	1
	25						30	1
	26						29	1
	27	S	M	02-Feb-93	Masaka		25	1
	28	M	M	01-Jul-74	Kicukiro		43	1
	29	B	M	15-Jun-80	Kicukiro		28	1
	31	J	W	23-Nov-92	Mohetetu		26	1
	32	J	M		Nyandarua		30	1
	37	T	S	15-Jun-93	Nakuru		25	1
	38	B	J	11-Nov-96	Starehe		22	0
	40	A	B	15-Jun-90	Kiambu		28	1
	41	M	M	15-Apr-86	Mombasa		31	1
	42	J	B	15-Jun-96				
	44	V	K	21-May-97			21	0
	45	C	J	20-Mar-96	Kisumu We		22	0
	46	V	S	09-Nov-98	Gatundu		20	0
	49	A	A	12-Dec-97	Kakamega		22	0
	50	R	M	28-Aug-97	Nairobi		22	0
	51	K	D	08-Jun-97	Kisii		21	0
	52	W	M		Machakos		21	0
	53	S	J	23-Aug-94	Kiambu		24	0

55	D	R	16-Aug-99	Nairobi	19	0
56	J	D	14-Mar-98	Starehe	20	0
57	P	M	15-Jun-90	Kisumu	28	1
58	C	M	21-Aug-92	Nakuru	26	1
59	A	S			22	0
61	B	M	02-Aug-92	Fizi	26	1
62	M	E		Kundu	21	0
63	C	N			24	0
65	A	R	07-Nov-96	Kiathi	22	0
68	A	R	15-Jun-97	Rukunjiri	21	0
69	P	K	06-Jul-94	Mwingi	24	0
70	K	E	15-Jun-84	Kiambu	34	1
71	H	J	07-Jul-84	Muranga	35	1
72	B	C	21-Nov-96	Huruma	22	0
73	C	P	11-Nov-93	Kampala	25	1
74	C	F	19-Nov-97	Siaya	21	0
75	H	M	19-Oct-89	Tala	30	1
76	B	A	25-Nov-97	Lurambi	22	0
77	E	E		Tanzania		
79	A	A	27-Dec-97		18	0
80	J	J	14-Sep-97	Machakos	22	0
81	A	B	20-Jun-85	Isingiro	33	1
83	S	A	22-Nov-00	Nairobi	18	0
84	B	M	04-Aug-96	Kamukunji	22	0
85	A	R	17-Dec-97	Nyeri	21	0
86	E	R	17-Dec-89	Rukungizi	29	1
88	S	E	12-Dec-97	Muranga	21	0
89	A	M	15-Jun-87	Machakos	31	1
90	J	F	01-Nov-75	Murang'a	43	1
91	M	R	12-Dec-96	Taita	22	0
92	K	N	18-Aug-96	Kampala	22	0
93	J	B	15-Jun-96	Ugenya	22	0
95	S	C	29-May-98	Bungoma	20	0
96	N	S	20-Jun-98	Kasarani	20	0
98	P	N		Mombasa	23	0
99	M	W	12-Oct-92	Vihiga	25	1
100	A	J	09-Feb-99	Runyenjes	19	0
101	J	S	15-Jun-92	Nairobi	27	1
102	A	R	15-Jun-99	Nairobi	19	0
103	V	H	28-May-96	Nairobi	21	0
104	S	A	15-Jun-92	Nairobi	27	1
105	P	J	02-Apr-88	Nairobi	30	1
106	A	A	15-Jun-93	Nairobi	26	1
107	A	P	15-Jun-99		19	0
108	A	C		Busia	23	0
109	D	N	15-Jun-91	Nairobi	28	1
110	V	V	15-Jun-96	Kisii	22	0
111	V	Q	15-Oct-96	Nairobi	22	0
112	I	B	18-Jun-83	Kakamega	35	1
113	J	M	07-Jun-94	Nairobi	22	0
114	P	S	15-Jun-90	Nairobi	29	1
115	S	B		Nairobi	22	0

117	Y	A	15-Jun-89	Nairobi	30	1
118	G	P	26-Jun-98	Kisii Cetral	20	0
119	P	V	15-Jun-99		19	0
120	D	A	25-Nov-96	Lurambi	23	0
121	T	A	15-Jun-88	Nairobi	31	1
123	P	S	15-Jun-92	Nairobi	26	1
124	S	W	15-Jun-87	Nairobi	32	1
125	M	R	12-Dec-98	Taita	22	0
126	D	C	15-Jun-86	Nairobi	33	1
127	J	J	25-Dec-97	Wareng	20	0
128	A	W	15-Jun-99		19	0
130	W	M	15-Jun-85	Nairobi	34	1
131	M	N	07-Mar-95	Kampala	23	0
132	J	L	15-Jun-96	Nairobi	22	0
133	E	W	11-Aug-98	Nairobi	20	0
135	L	M	15-Jun-96	Nairobi	22	0
136	S	W	15-Jun-84	Nairobi	35	1
138	M	E		Kisumu	22	0
139	K	P	15-Jun-85	Nairobi	34	1
140	C	D	15-Jun-87	Siaya	31	1
141	B	V	15-Jun-94	Kakamega	25	1
142	P	V	30-Sep-90	Kajiado	24	0
143	C	P	15-Jun-88	Nairobi	31	1
145	M	E	27-Aug-99	Embu	19	0
146	A	M	15-Jun-99		19	0
147	T	M	01-Jan-85	Rachuonyo	38	1
148	A	L	12-Dec-96	Kigali	22	0
149	G	S	15-Jun-99		19	0
150	K	J	06-Dec-94	Kirinyaga	27	1
153	A	M	19-Dec-96	Kinangop	22	0
154	N	M	08-Aug-90	Kiambu	28	1
157	J	S	10-Oct-91	Kiambu	27	1
163	E	A	10-Oct-97	Kasarani	22	0
166	J	L	25-Dec-95	Thika	23	0
173	P	M	02-Jun-89	Nairobi	29	1
174	P		15-Jun-92	Embu	27	1
177	V					
179	S	M	17-Aug-83	Kandara	35	1
181	K	F	15-Jun-90	Murang'a	28	1
184	E	K	24-Oct-91	Kiambu	27	1
185	M	E	23-Feb-00	Nakuru	18	0
186	B	J	07-Jan-18	Kiambu	18	0
189	C	M	08-Oct-00	Kiambu	18	0
190	E	E	03-Sep-97	Kiambu	21	0
191	H	M	20-Oct-97	Kiambu	21	0
194	J	M	15-Jun-96	Murang'a	22	0
196	B	S	08-Aug-96	Githurai	22	0
197					24	0
198	F	H	29-Jan-93	Githurai	24	0
200		M	12-Sep-98	Kiambu	20	0
202	D	M	19-Jul-98		20	0
204	E				21	0

	205	B	K	12-Jun-95	Kaspul	23	0
	206	S	K				
	209	K		15-Jun-92	Kiambu	25	1
	210	B	N	15-Jun-96	Kiambu	23	0
	211	D		09-Aug-88			
	212	J				21	0
	213	Z	C	01-Apr-96	Bungoma	23	0
	214	D	H	18-Nov-80	Mombasa	38	1
	216	C	R	15-Dec-89	Busia	30	1
	221	A				21	0
	224	J				22	0
	229					28	1
	230	F	Y	15-Jun-82		36	1
	231	J	M	15-Jun-97	Nakuru	21	0
	232	D	E	08-Mar-98	Mombasa		
	233	B	E	15-Jun-97	Kamwangi	21	0
	234	M	J			20	0
	235	W	M		Gatundu	19	0
	238	S	A		Githurai	19	0
	239	H	F		Githurai	20	0
	240	S	E	15-Jun-99	Githurai	20	0
	244						
	246	C	M				
	248	J	H	09-Jun-98	Ruiru	20	0
	249	S	S	15-Jun-93	Kiambu	25	1
	251	N	W	07-Aug-94		24	0
	252	N	S	20-Feb-95	Nakuru	22	0
	253	M	M			20	0
	254	J		06-Mar-82	Thika	36	1
	255				Kiambu	24	0
	256	N	N	04-Aug-90			
	259	A	W	04-Sep-00	Kiambu	19	0
	261	S	L	16-Jan-99	Gatundu	19	0
	262	Y	Z	15-Nov-90	Miritini	28	1
	263	J	C	20-Dec-94	Nairobi	24	0
	264		N	20-Apr-96	Kiambu	22	0
	267	D		01-Sep-94	Nairobi	28	1
	269	D	A	20-Jan-98	Nairobi	20	0
	270	F	C	27-Nov-98	Nairobi	20	0
	272	C	I	25-Dec-98	Nairobi	20	0
	274	L	G	20-Jan-96	Baringo	22	0
275a	275	W	R	15-Jun-98	Thika	24	0
	276	G	R	11-Dec-95	Nairobi	23	0
	280	S	J	15-Jun-92	Bahati	26	1
	290	B		15-Jun-95	Kiambu	22	0
	293	B	C	15-Jun-95	Pumwani	23	0
	294	F	G	15-Jun-78	Kiambu	38	1
	295	P	M	10-Oct-99	Githurai	19	0
	296			08-Oct-86			
	297		R	15-Jun-91	Ukinda	28	1
	300	B	N	25-May-87	Kiambu	29	1
	301	E	R	08-Aug-96	Kisii	23	0

302	R	P	21-Aug-96	Kisumu	22	0
303		N		Kiambu		
304	C	E	15-Jun-92	Gatanga	26	1
305	V	I	09-Nov-98	Kimbo	20	0
306	D	R	16-Aug-99		19	0
308	A	E	12-Dec-97		21	0
309	N	A	17-Mar-95	Githurai	23	0
312	D	M	18-Jul-00	Nairobi	18	0
314	C	h	15-Jun-98	Mathare	20	0
315	L	H	28-May-96	Pumwani	21	0
316	S	N	25-May-95	Nyeri	25	1
319	C	J	12-Nov-96	Kiambu	25	1
320	J	J	12-Apr-96		25	1
322	J	W	15-Jun-96		22	0
323	M	R	15-Jun-89	Vihiga	30	1
325				Kiambu		
326				Kiambu		
332	B	M	15-Jun-95	Pumwani	23	0
333	M	O		Ruaraka		
335	N	N		Kiriaini	20	0
336	G	N	15-Jun-94	Kiambu	25	1
337	D	F	10-Mar-00	Gatundu	18	0
338	S	E	22-Nov-94	Kiambu	24	0
340	E	J	01-Aug-97	Makadara	21	0
342	S	E	15-Jun-94	Nairobi	24	0
344	W	J	14-Sep-98	Bungoma	23	0
346	B	C		Thika	23	0
348	B	C	09-Sep-95	Dandora	22	0
351						
352	K	A	02-Dec-99	Gatundu	19	0
355	S	M	06-Aug-93	Nairobi	25	1
356	I	J	22-Apr-86	Gatundu N	32	1
357	A	J	20-Feb-82	Garsen	36	1
358	K	M	15-Jun-90	Murang'a		
360	J	W	21-Mar-99	Embakasi N	19	0
361	T	M			21	0
362	F	M	13-Jul-95	Tetu	23	0
363	A				24	0
364	O	J	24-Apr-85	Thika	32	1
365	J				24	0
366		V	14-Sep-99	Nanyuki	19	0
368	P	L	16-May-99	Thika	19	0
369	B	F	19-Feb-93	Nairobi	25	1
374	V	T	15-Jun-99	Nairobi	19	0
375	H	E	12-Dec-95	Kisumu	23	0
3	K	S	05-Sep-94	Gatundu North		
4	F	I	26-Nov-96	Kakamega E	21	0
7	A	C	01-Jun-96	Kakamega	22	0
13	S	T	10-Apr-91	Kisii	28	1
16	J	R	15-Jun-91	Kapenguria	27	1
23	K	L	25-Oct-95	Makindie	23	0
30	F	B	04-Apr-91	Kampala	27	1

33	P	R	03-Mar-89	Vihiga	29	1
34	G	M	17-Jan-84	Kitui	34	1
35	D	M	25-Feb-96	Busia	21	0
36	F	F	21-Sep-98	Kitui		
39	J	J	14-Sep-97	Makueni	22	0
43	J	M	02-Aug-97	Kiambaa	21	0
47	N	N	15-Jun-95	Moshi	24	0
48	B	T	20-Jul-98	Mchakos	20	0
54	J	H	10-Aug-94	Nakuru	24	0
60	M	K		Namutumb	23	0
64	E	W	02-Oct-94	Mombasa	24	0
66	N	M	16-Jun-90	Nakuru	28	1
67	H	N	21-Oct-92		26	1
78	O	R	30-Apr-99	Kangondo	20	0
82	M	K	15-Jun-90	Migori	28	1
87	S	J	15-Jun-96	Sergo	22	0
94	M	N	20-Nov-79	Kiambu	39	1
97	C	M	14-May-95	Nyeri	23	0
116	S	C	19-May-98	Bungoma	20	0
122	C	F	22-Sep-79	Omu-Aran-I	39	1
129	O	T	30-May-93	Igara	25	1
134	J	A	05-Jun-91	Machakos	27	1
144	M	B	11-Dec-81	Murang'a	37	1
152	S		08-Dec-94	Kiambu	25	1
155					22	0
156	G	M	14-Aug-97	Kiambu	21	0
160					28	1
161	V	W	29-Oct-99	Nairobi	19	0
162	F	P	12-Dec-99	Kwale	20	0
164				Kisumu	23	0
165	K	P				
167	S	G	28-Dec-00	Machakos	18	0
168	C	E	20-Oct-88	Bungoma	29	1
169	F	A	11-Apr-97	Kiambu	21	0
172	D	L	15-Jun-94	Lari	29	1
175	F	C	15-Jun-95	Siaya	24	0
176	P	S	08-May-96	Murang'a	24	0
178	D	M	15-Jun-94	Roysambu	24	0
180	C	D			23	0
182	B	F	14-Mar-97		21	0
187	J	J	09-Feb-93		25	1
188	B	G				
192	I	M	15-Jun-96	Nairobi	24	0
195	F	C	30-Apr-94	Kasarani	24	0
199	O	C	18-Apr-98	Kiambu	21	0
201						
203				Kilifi	20	0
208	H				23	0
219	J	M		Kiambu		
225	F	J	15-Jun-97	Kisii	21	0
226	R	P	15-Jun-98	Embakasi	19	0
227	S	E	28-Mar-97	Kiambu	21	0

228	K	N	16-Apr-99	Nairobi	19	0
236	A	N	30-Sep-98	Embu	20	0
237	M	F	15-Jun-97	Nakuru	21	0
241	J	M	15-Jun-95	Nairobi	23	0
243	N	J	15-Jun-90			
245	K	G	12-Jan-98	Ruiru	20	0
247	D	M			21	0
250	S	J	19-Dec-99	Nyeri	18	0
257	M	M		Thika	23	0
258	P	N	15-Jun-66	Thika	49	1
260	S	F	15-Jun-99	Thika	19	0
265	Y				28	1
266				Nairobi	22	0
268	C	L				
271	C	M	28-Mar-98	Nairobi	20	0
273	A	M	02-Jun-85	Nairobi	33	1
275	P	J	03-Nov-93	Kakamega	26	1
277	C	S	12-Jan-85	Kirinyaga	33	1
278	S	R	22-Mar-94	Kiambu	24	0
279	J	D				
289	K	D	15-Aug-87	Khwisero	31	1
291	A	R	03-Nov-96	kakamega	23	0
292	E	A	16-Jun-97	Siaya	23	0
298	D	R	07-Aug-00	Githurai	18	0
299	B	M	05-Apr-93	Starehe	25	1
307	M	E	25-Sep-98	Mtwapa	20	0
310	T	Z	15-Jun-86	Nairobi	32	1
311	R	W	15-Jun-95	Nairobi	23	0
313	A	A	02-May-92	Kirinyaga	26	1
317			15-Jun-96	Nyeri		
318	F	M	26-Sep-97		21	0
321	M	E	28-Jul-99	Nairobi	19	0
324	W	J	14-Sep-98	Bungoma	23	0
327	J	E	03-Apr-98	Githurai	21	0
328	J	W	08-Jan-94	Ikinu	27	1
329						
330	J	F		Ruiru		
334	J	W		Ruiru	23	0
339					24	0
341	K	M	17-Feb-96	Thika	22	0
343	W	J	15-Oct-98		20	0
347	L	C	20-Jun-96	Embakasi	20	0
349	C	M	15-Jun-98	Ougadoudc	20	0
350	S	G	15-Jun-93	Kiambu	25	1
354	T	P	29-Oct-93	Kericho	24	0
359	K	J	15-Jun-94	Kiambu	24	0
367	E	M	18-Nov-90	Kiambu	27	1
370	P	R	13-Oct-94	Thika	24	0
371	M	C	17-Feb-96	Thika	22	0
372					23	0
373					25	1
137	B	J	12-Aug-93	Nairobi	24	0

215	N	M	05-Apr-88	Mumias	29	1
217	D	W	15-Jun-96	Bungoma	23	0
220	F	L	15-Jun-98	Kiambu	20	0
223	F				21	0
331	D	W	20-Jan-98			
21	E	O	08-Feb-96	Kendu bay	22	0
183	J	T	06-Dec-93	Nairobi	25	1
193	V	P	02-Aug-98	Embu	24	0
345	A			Nakuru	22	0

Q102	Q102REC	Q103	Q104	Q104REC	Others	Q105	Q105REC	Q106
Place of Birth	Place of Birth	Place of Residence	Religion	Religion	If other specify	Education level	Education level	Sexually dentity impacted education
1	0.00	Githiga	2	0		3	0	0
1		Githunguri	4	1		3	0	0
1	0.00	Githungi	2	0		3	0	0
1		Ruiru	1	0		4	1	0
1		Kiambu	2	0		2	0	0
1	0.00	Kiambu	1	0		2	0	0
1	0.00	Ruaka	7	1		2	0	0
1		Githurai	7	1		3	0	0
1	1.00	Donholm	1	0		4	1	
1	0.00	Zimerman	3	1		3	0	1
1	0.00	Kiserian	1	0		4	1	0
1	0.00	Nairobi	1	0		4	1	0
1	0.00	Nairobi	1	0		3	0	1
1	0.00	Nairobi	1	0		4	1	0
1		Nyani	1	0		4	1	1
2	0.00	South B	1	0		4	1	0
1	0.00	Nairobi	1	0		3	0	0
2	0.00	Estleigh	4	1		3	0	0
1	0.00	Roysambu	1	0		4	1	0
1	0.00	Nairobi	3	1		4	1	0
1	0.00	Kangemi	1	0		3	0	1
1	0.00	Nairobi	7	1		3	0	0
1	0.00	Amboseli	2	0		3	0	1
2	0.00	Muthama	1	0		3	0	0
3	0.00	Nairobi	1	0		4	1	1
1	0.00	Kiambu	7	1		3	0	
	0.00		2	0		3	0	
2	0.00	Donholm	1	0		4	1	1
2	0.00	Kabiria	1	0		3	0	1
4	1.00	Nairobi	2	0		4	1	0
4	0.00	Kasarani	2	0		3	0	1
1	1.00	Majengo	1	0		3	0	1
1	0.00	Nairobi	2	0		4	1	
1	0.00	Nairobi	1	0		4	1	0
1	0.00	Huruma	7	1		4	1	0
1	1.00	Nairobi	1	0		3	0	0
1	1.00	Nairobi	3	1		4	1	1
	0.00		2	0		4	1	0
	1.00	Gethoria	1	0		3	0	
1	0.00	Kasarani	2	0		4	1	0
1	0.00	Thika	1	0		3	0	0
1	0.00	Saika	1	0		4	1	0
1	1.00	Nairobi	1	0		3	0	0
1	0.00	Ngara	1	0		3	0	0
1	0.00	Machakos	1	0		3	0	1
1	0.00	Niarobi	1	0		3	0	0

1	0.00	Mathare	1	0	2	0	0
1	0.00	Huruma	7	1	3	0	0
1	1.00	Nairobi	1	0	2	0	1
1	0.00	Nairobi	7	1	2	0	0
2	0.00		3	1	2	0	1
3	0.00	Umoja	2	0	4	1	0
3	0.00	Umoja	2	0	3	0	0
3	0.00	Umoja	2	0	4	1	0
4	0.00	Kasarani	2	0	4	1	0
2	1.00	Rongai	1	0	4	1	1
1	0.00	Huruma	1	0	4	1	0
1	0.00	Uthiru	2	0	3	0	0
1	0.00	South B	2	0	3	0	0
1	0.00	Huruma	2	0	3	0	0
2	0.00	Pangani	3	1	4	1	1
1	0.00	Kawangware	2	0	3	0	1
1	0.00	Huruma	1	0	2	0	0
1	0.00	Huruma	2	0	3	0	0
5	0.00	Ruaka	1	0	3	0	0
1	0.00	Mlango Kubw	3	1	2	0	1
1	0.00	Nairobi	1	0	4	1	0
1	0.00	Nairobi	1	0	3	0	1
1	0.00	Kariobang'i	3	1	3	0	0
1	1.00	Kasarani	7	1	3	0	0
1	0.00	Nairobi	2	0	3	0	0
2	0.00	Eastleigh	1	0	2	0	1
1	0.00	Baba dogo	2	0	3	0	0
1	0.00	Mlango	1	0	3	0	0
1	0.00	Ngara	1	0	3	0	0
1	0.00	Nairobi	3	1	3	0	0
1	0.00	Nairobi	3	1	3	0	1
1	0.00	Huruma	1	0	3	0	0
1	0.00	Nairobi	1	0	3	0	0
1	0.00	Nairobi	2	0	4	1	0
1	0.00	Nairobi	1	0	3	0	0
1	0.00	Huruma	1	0	3	0	0
1	0.00	Nairobi	2	0	3	0	0
1	0.00	CBD	2	0	4	1	0
1	0.00	Majengo	3	1	2	0	0
1	0.00	Huruma	1	0	3	0	0
1	0.00	CBD	1	0	4	1	0
1	1.00	Kariobangi	2	0	3	0	0
1	0.00	CBD	2	0	4	1	0
	0.00		3	1	2	0	0
1	1.00	Kasarani	1	0	4	1	0
1	0.00	CBD	2	0	4	1	0
1	0.00	Kance	2	0	2	0	0
1	0.00	Kikuyu	3	1	3	0	0
1	0.00	Ruaraka	2	0	3	0	0
1	0.00	Kahawa Wend	1	0	3	0	1
1	0.00	CBD	1	0	4	1	0
1	0.00	Kikuyu	3	1	3	0	0

1	0.00	CBD	2	0	3	0	0
1	0.00	Town Center	7	1	3	0	0
1	0.00	Kariakor	3	1	2	0	0
1	0.00	Huruma	2	0	3	0	0
1	0.00	CBD	1	0	3	0	0
1	0.00	Westlands	3	1	3	0	0
1	0.00	CBD	2	0	3	0	0
1	1.00	Nairobi	3	1	3	0	0
1	0.00	CBD	2	0	3	0	0
1	0.00	Ng'ara	1	0	3	0	0
1	0.00	Majengo	3	1	2	0	0
1	0.00	CBD	1	0	4	1	0
2	0.00	Matasia -Ngoı	3	1	4	1	0
1	0.00	Kikuyu	2	0	3	0	0
1	0.00	Huruma	3	1	3	0	0
1	0.00	Kikuyu	3	1	3	0	0
1	0.00	CBD	1	0	4	1	0
1	0.00	Huruma	1	0	3	0	0
1	0.00	CBD	1	0	4	1	0
1	1.00	Majengo	1	0	2	0	0
1	0.00	Nairobi	4	1	2	0	1
1	0.00	Nyeri	6	1	4	1	0
1	0.00	CBD	2	0	4	1	0
1	0.00	Karen	1	0	3	0	0
1	0.00	Majengo	3	1	2	0	0
1	0.00	Majengo	2	0	3	0	0
4	0.00	Kasarani	1	0	3	0	0
1	0.00	Majengo	3	1	2	0	0
1	0.00	Juja	1	0	3	0	0
1	0.00		4	1	3	0	1
1	1.00	Kiambu	4	1	4	1	0
1	1.00	Kiambu	3	1	2	0	1
1	0.00	Nairobi	1	0	4	1	0
1	0.00	Gatukuyu	2	0	3	0	
1	0.00	Githurai	2	0	2	0	0
1	0.00	Embu	1	0	3	0	0
	0.00		1	0	3	0	1
1	0.00	Githurai	2	0	3	0	0
1	0.00	Githurai	1	0	3	0	0
1	0.00	Kiambu	1	0	3	0	0
1	0.00	Githurai	1	0	3	0	0
1	0.00	Kiambu	7	1	3	0	0
1	0.00	Kiambu	1	0	3	0	0
1	0.00	Kiambu	1	0	3	0	0
1	0.00	Kiambu	1	0	3	0	0
1	0.00	Githurai	2	0	3	0	0
1	0.00	Githurai	4	1	4	1	0
1	0.00	Juja	1	0	3	0	0
1	0.00	Githurai	3	1	4	1	0
1	0.00	Githurai	3	1	4	1	0
1	1.00	Nairobi	7	1	4	1	0
1	0.00	Nairobi	1	0	4	1	0

1	0.00	Githurai	7	1	3	0	0
1	0.00	Ruaka	2	0	3	0	0
1	0.00	Ruaka	1	0	4	1	1
1	0.00	Muchatha	4	1	2	0	0
	0.00		2	0	1	0	0
1	0.00	Ruaka	2	0	3	0	0
1	0.00	Bungoma	1	0	3	0	0
1	0.00	Muchatha	1	0	3	0	1
1	0.00	Kiambu	1	0	2	0	0
1	1.00	Ruaka	1	0	3	0	0
1	0.00		1	0	4	1	0
1	0.00	Githurai	2	0	3	0	0
1			7	1	3	0	0
1	0.00	Nairobi	2	0	4	1	0
	0.00		1	0	3	0	0
1	0.00	Thika	1	0	4	1	0
1	0.00	Ruiru	3	1	4	1	1
1	0.00	Ruiru	1	0	4	1	1
1	0.00	Githurai	1	0	4	1	0
1	1.00	Githurai 45	3	1	3	0	0
1	0.00	Githurai 45	1	0	3	0	1
	0.00		1	0	4	1	0
	0.00		1	0	4	1	0
1	0.00	Ruiru	2	0	3	0	1
1	0.00	Ruiru	1	0	4	1	0
1	0.00	Thika	7	1	4	1	0
1	0.00	Ruaka	1	0	4	1	0
1	0.00	Nairobi	3	1	4	1	0
1	0.00	Mangui	1	0	4	1	0
1	0.00	Thika	7	1	4	1	1
	0.00						
1	0.00	Thika	2	0	4	1	
1	0.00	Thika	7	1	4	1	
1	0.00	Githurai	3	1	3	0	0
1	0.00	Githurai	1	0	2	0	0
1	0.00	Pipeline	7	1	4	1	0
1	0.00	Githurai	7	1	4	1	
1	0.00	Githurai	1	0	3	0	0
1	0.00	Githurai Kmb	1	0	3	0	0
1	0.00	Githurai	7	1	4	1	0
1	0.00	Kasarani	7	1	4	1	0
1	1.00	Thika	5	1	3	0	1
1	1.00	Githurai	1	0	3	0	0
1	0.00	Githurai	4	1	2	0	0
1	0.00	Babadogo	7	1	4	1	0
1	0.00	Dandora	7	1	2	0	0
1	1.00	Nairobi	2	0	4	1	0
1	1.00	Githurai Kiml	7	1	3	0	0
1	0.00	Nairobi	1	0	3	0	0
1	0.00		3	1	4	1	0
1	0.00	Kiambu	2	0	4	1	0
1	0.00	Kahawa West	1	0	4	1	0

1	0.00	Kasarani	7	1	4	1	0
	1.00	Kiambu	3	1	2	0	
1	0.00	Thika	1	0	4	1	0
1	0.00	Kiambu	1	0	3	0	0
1	0.00	Nairobi	1	0	1	0	0
1	0.00	Githurai	2	0	3	0	0
1	0.00	Githurai	1	0	3	0	0
1	0.00	Githurai	7	1	1	0	0
1	0.00	Huruma	2	0	3	0	1
1	0.00	Githurai	1	0	3	0	0
1	0.00	Mwiki	1	0	2	0	0
1	0.00	Kiambu	2	0	4	1	0
1	0.00	Kiambu	1	0	4	1	0
1	0.00	Kikuyu	1	0	3	0	0
1	0.00		4	1	3	0	
1	0.00	Kiambu	3	1	3	0	0
1	1.00	Kiambu	4	1	3	0	1
1	0.00	Dandora	7	1	3	0	0
1	0.00		1	0	3	0	1
1	0.00	Maragua	4	1	4	1	0
1	1.00	Juja	2	0	3	0	0
1	0.00	Nairobi	2	0	4	1	0
1	0.00	Ruiru	1	0	3	0	0
1	0.00	Juja	1	0	4	1	0
1	1.00	Ruiru	1	0	4	1	0
1	0.00	Juja	2	0	4	1	0
1	0.00	Juja	2	0	4	1	0
1	0.00	Pumwani	7	1	3	0	0
1	0.00	Muthiga	1	0	3	0	0
1	0.00	Thika	2	0	4	1	0
1	0.00	Gatundu	1	0	4	1	0
1	0.00	Kiambu	3	1	2	0	0
1	0.00	Thika	1	0	3	0	0
1	0.00	Thika	1	0	4	1	0
1	0.00	Thika	7	1	1	0	0
1	0.00	Githurai	1	0	2	0	0
1	0.00	Ruaka	2	0	4	1	0
1	1.00	Thika	1	0	3	0	1
1	0.00		1	0	3	0	
1	0.00		1	0	4	1	0
1	1.00	Thika	2	0	4	1	0
1	0.00	Amboseli	2	0	4	1	0
1	0.00	Nairobi	1	0	4	1	0
1	0.00	Kasarani	2	0	4	1	0
1	0.00	Nairobi	1	0	3	0	1
1	0.00		1	0	3	0	0
1	0.00	Nairobi	1	0	3	0	0
1	1.00		3	1	4	1	0
1	0.00	Nairobi	1	0	3	0	0
2	0.00	Muthama	1	0	3	0	1
2	0.00	Kampala	3	1	3	0	0

1	1.00	Kasarani	1	0	3	0	0
1	1.00	Nairobi	7	1	4	1	1
1	0.00	Karibangi Sou	1	0	2	0	0
1	0.00	Ongata Ronga	2	0	3	0	0
1	0.00	Nairobi	2	0	4	1	0
1	0.00	Ruaka	1	0	3	0	0
5	0.00	Juja	3	1	4	1	0
1	0.00	Buruburu	2	0	4	1	0
1	1.00	Westlands	2	0	3	0	1
2	0.00	Joska	2	0	3	0	1
1	0.00	Rongai	1	0	3	0	0
1	0.00	Rongai	1	0	3	0	0
1	0.00	South B	3	1	3	0	1
1	0.00	Huruma	2	0	3	0	0
1	0.00	Pangani	1	0	4	1	0
1	0.00	Nairobi			3	0	0
1		Nairobi	2	0	3	0	1
1	0.00	Mwiki	3	1	4	1	0
1	0.00	Pangani	1	0	3	0	0
6		Kabiria	1	0	4	1	
2	0.00	Eastleigh	1	0	3	0	1
1	0.00	Alsops	1	0	3	0	0
1	1.00	Githurai 45	1	0	3	0	0
1	0.00	Githurai	1	0	3	0	0
1	0.00	Kiambu	2	0	3	0	0
1	0.00	Kinoo	1	0	3	0	0
1	1.00	Githunguri	2	0	3	0	0
1	0.00	Ruiru Kimbo	1	0	4	1	
1	0.00	Nairobi	1	0	4	1	0
1	0.00		7	1	4	1	0
1	0.00	Nairobi	2	0	4	1	0
1	0.00	Kenyatta Roa	2	0	3	0	0
1	0.00	Kimemnde	7	1	3	0	0
1	0.00	Kiambu	1	0	3	0	0
1	0.00	Nairobi	2	0	4	1	0
1	0.00	Githurai	1	0	3	0	0
1	0.00	Githurai	7	1	3	0	0
1	0.00	Kasarani	3	1	3	0	0
1	0.00	Nairobi	1	0	4	1	0
1	0.00	Kirinyaga	1	0	3	0	0
1	0.00	Githurai	7	1	3	0	0
1	0.00	Githurai	1	0	3	0	1
1	0.00	Ruaraka	1	0	4	1	0
1	0.00	Githurai	2	0	4	1	0
	1.00		1	0	3	0	
1	0.00	Ruai	2	0	4	1	0
1	0.00	Ruaka	7	1	3	0	0
1	0.00	Kiambu	1	0	1	0	0
1	0.00				4	1	0
1	0.00	Githurai 45	3	1	4	1	0
1	0.00	Nairobi	2	0	4	1	0

1	0.00	Kiambu	1	0	3	0	
1	0.00	Nairobi	2	0	4	1	0
1	0.00		2	0	4	1	1
1	0.00	Mwiki	7	1	4	1	0
1	0.00		2	0			
1	0.00		2	0	4	1	0
1	0.00	Ruiru	2	0	4	1	0
1	0.00		2	0	4	1	0
1	0.00	Juja	2	0	4	1	0
1	1.00	Thika	1	0	4	1	0
1	0.00	Thika	2	0	4	1	0
1	1.00	Githurai	3	1	4	1	0
1	1.00	Githurai	3	1	4	1	0
1	0.00		3	1	3	0	1
1	0.00	Githurai	1	0	3	0	0
1	0.00	Zimmerman	2	0	3	0	0
1	0.00	Kawangware	1	0	3	0	
1	1.00	Nairobi	2	0	4	1	0
1	0.00	Githurai	1	0	4	1	0
			4	1	3	0	0
1	1.00	Githurai	7	1	3	0	0
1	0.00	Umoja	1	0	4	1	0
1	0.00	Muthiga	1	0	4	1	1
1	0.00	Githurai	1	0	3	0	0
1	0.00	Githurai	2	0	4	1	0
1	0.00	Nairobi	7	1	4	1	0
1	1.00	Kasarani	3	1	4	1	
1	0.00	Githurai	1	0	4	1	0
1	1.00	Kiambu	7	1	3	0	0
1	1.00	Githurai	4	1	2	0	0
1	0.00		1	0	4	1	0
1	0.00	Roysambu	2	0	3	0	0
1	0.00	Juja	2	0	4	1	0
1	0.00	Githurai	7	1	3	0	0
1	1.00	Kiambu	2	0	3	0	0
			1	0	3	0	0
	1.00		2	0	3	0	1
1		Kenyatta Roa	1	0	3	0	0
1	0.00	Kiambu	1	0	3	0	0
1	0.00	Thika	1	0	4	1	0
1	0.00		7	1	4	1	0
1	0.00	Umoja	2	0	4	1	0
7	0.00		2	0	4	1	0
1	0.00	Kiambu	1	0	3	0	1
1	0.00	Thika	2	0	4	1	0
1	0.00	Thika	1	0	3	0	0
1	0.00	Thika	2	0	4	1	0
1	0.00	Makongeni	2	0	4	1	0
1	0.00	Thika	1	0	4	1	0
1	0.00	Thika	1	0	3	0	0
1	1.00	Githurai	1	0	3	0	0
1	1.00	Nairobi	1	0	2	0	0

1	0.00	Ruaka	1	0	2	0	0
1	0.00	Ruaka	1	0	3	0	0
1	0.00	Ruaka	1	0	3	0	0
1	0.00	Ruaka	2	0	3	0	
	0.00		7	1	2	0	0
1	0.00	Seka	3	1	3	0	0
1	0.00	Kasarani	1	0	3	0	0
1	0.00	Githurai	1	0	3	0	0
1	0.00	Juja	2	0	4	1	0

Ifyeshow	Q107	If_yesa	If_yesaREC	Incomeb	IncomebRe	Q108	IfYes	Q109
If yes how	Currently employed	If yes	If Currently employed	Monthly income	Monthly income	Identify as MSM	If yes how long MSM	Identity MSW
	0							
	1	1	0					
		3	1			1		1
	0							0
	0					0		0
	0			1	0	0		0
	0			1	0	0		0
	0							
	1							
able to surv	1	2	1	1	0	1	8 Years	1
	1	3	1	3	1	1		1
	1	1	0	3	1	1	10 years	1
	1			1	0	1		1
	1	2	1	2	1	1	12 Years	1
Discriminat	0					1	8 Years	1
	1	2	1	1	0	1		0
		2	1	1	0	1	20 Years	1
	0			1	0	1	5 Years	1
	0					1	5 Years	1
	1	3	1	3	1	1	Long	1
Dropped ou	0	1	0	1	0	1	10 Years	1
				1	0	1	6 Years	1
Suspenden	1	3	1	1	0	1	5 Years	1
	0	1	0	1	0	1		0
	0					1	13 Years	0
	1	1	0	2	1	1	7 Years	1
	1	1	0	1	0	1		1
		2	1	1	0	1	16 Years	
Suspenden	0			1	0	1		0
	1	3	1	2	1	1	22 Years	0
	0					1		1
				3	1	1		1
Suspended	1	1	0	3	1	1	All life	0
	1	1	0	1	0	1		0
	0					1		1
	1	1	0	2	1	1	10 Years	0
	0			2	1	0		1
	1	1	0	3	1	1	3 Years	
						1	4 Years	0
	0					1	3 Years	
		4	1			1	1 Year	0
		2	1			1	3 Years	0
	1	1	0	1	0	1		1
	1	2	1	1	0	1		1
		4	1	1	0	1		1
	1	2	1	1	0	1	4 Years	1

	0			1	0	1		1
	0					1		1
	1	1	0	2	1	1	8 Years	1
	0	4	1	1	0	1	7 Years	1
				1	0		3 Years	1
	0					1		1
	0					1	1 Year	1
	0					1		1
	0					1	2 Years	1
Teased abo	1	2	1	1	0	1	Childhood	1
	0					1	4 Years	1
	0	4	1			1	10 Yrs	1
	0					1	10 Years	1
	0					1	5 Years	0
Homophob	1	2	1	2	1	1	25 Years	0
Paid off par	0					0		
	1	2	1	1	0	1	16 Years	1
	0					1	4 Years	0
	0			1	0	1		1
	0			1	0	1	6 Years	1
		1	0	2	1	1		1
Expelled from school		4	1			1	13 years	0
	0					1		1
	1	2	1	2	1	1	7 Years	0
	1	3	1	2	1	1	12 Years	1
Rejected to	0	4	1	1	0	1		1
	0					1	5 Years	0
	0	2	1	4	1	1		0
	0					1		1
	0					1	6 Years	0
	0			1	0	1		
	1	2	1	1	0	1	4 Years	1
	1	2	1	1	0	1	3 Years	0
		4	1			1		0
	1			1	0	1	3 Years	1
	1	2	1	2	1	1	3 Years	1
	0					1	2 Years	1
	1	1	0	2	1	1		1
	0					1		1
	0			1	0	1	6 Years	1
	1	1	0	2	1	1		1
	1	3	1	2	1	1	All my life	0
	1	1	0	2	1	1		1
	0					1		1
	0					1	4 Years	0
	1	2	1	2	1	1		1
	1	2	1	1	0	1	1 Year	1
	0					1		0
	1	2	1	2	1	1		1
I have know	1	1	0	2	1	1	5 Years	1
	1	2	1	2	1	1		1
	0					1		0

1	1	0	2	1	1		1
0					1	5 Years	1
0					1		1
0					1		0
0	2	1	2	1	1		1
0					1	10 Years	0
1	2	1	2	1	1		1
0					1	6 Years	1
1	1	0	2	1	1		1
	3	1	2	1	1	6 Years	1
0					1		1
1	2	1	2	1	1		1
0					1	5 Years	1
0					1		0
1	2	1	1	0	1	5 Years	1
0					1		0
1	1	0	2	1	1		1
0			1	0	1	5 Years	1
1	2	1	2	1	1		1
0			2	1	1	6 Years	1
1	1	0	1	0	1		1
1	2	1	2	1	1		0
1	1	0	2	1	1		1
0					1	1 Year	1
0					1		1
0			1	0	1	15 Years	1
0					1	3 Years	1
0					1		1
	3	1	2	1	0		1
1	3	1	1	0	1		1
1	3	1	2	1	1	4 Years	0
0	3	1	1	0	1		1
0					1		0
0					1		0
1	1	0	2	1	1	5 Years	0
0			3	1	0		1
0			3	1	0		0
0					1	25 - 30 Year	0
1					1		0
1	1	0	2	1	1	7 Years	0
0					1		
0					1	2 Years	1
0					1		0
0					1	2 Years	0
0					1	3 Years	0
0					1		1
0					1	3 Years	1
0					1		0
0					1	1 Year	1
0	1	0	1	0	1	7 Years	0
0					1	2 Years	1
0					1	2 Years	0

	0					0		0
	0			2	1	1		1
	1			1	0	1		1
	0			1	0	1	2 Years	1
	0			1	0	1	2 Years	1
	1	2	1	2	1	1		1
	0			1	0	1		1
People usec	0	4	1	1	0	1	20 Years	0
	0			1	0	1	2 Years	1
		1	0	1	0	0		0
	0	2	1	2	1	1		1
	0	4	1			1	10 Years	0
	0	4	1					
	0							0
	0							
	1	2	1	1	0	1		1
	0					1	3 Years	0
Masoko	0					1	4 Years	0
	0					1		0
	0					1	2 Years	0
	1	2	1	1	0	1	1 Year	1
	1	2	1	1	0	1		0
	1			2	1	1		0
		4	1	1	0	1		0
	0					0		0
	0							
	0	4	1	1	0	1		1
	0			1	0	1	6 Years	0
	1	1	0	2	1	1	1 Year	0
	0					1	3 Years	0
						1		0
		2	1	2	1	1	6 Years	0
	0					1	4 Years	0
	1	1	0	2	1	1	6 Years	0
	0			1	0	0		0
	1	3	1	3	1	1	3 Years	0
	1	4	1	2	1	1		0
	1	3	1	1	0	0		0
	0			1	0	1	2 years	1
	0					1	3 Years	1
	0					1	6 Years	0
	0	4	1			1	12 Years	1
No how to l	0	4	1	1	0	1	4 Years	1
	1					1		0
	0			1	0	1	3 Years	
		4	1			1	6 Years	1
	1	1	0	3	1	1	14 Years	0
	1	2	1	2	1	1		0
	0	4	1			1	3 Years	0
	1	3	1	1	0	1	10 Years	1
	1	2	1	3	1	1		0
	1	1	0	3	1	1	8 Years	1

	0					1	2 Years	0
		2	1	1	0	1		1
	1			2	1	1	Since birth	0
	0	4	1			1	10 Years	1
	1	3	1	1	0	1	2 Years	1
Kept it secr	0			2	1	1	3 Year	1
	0	4	1			1		1
						1	4 Years	1
	0	1	0	1	0	1	4 Years	1
	0					1	3 Years	1
	0	4	1	2	1	1	6 Years	1
	1	1	0	4	1	1	2 Years	0
	1	1	0	4	1	1		0
	1	3	1			1	2 Years	0
		1	0	2	1	1	3 Years	0
	0					1	2 Years	1
	1	3	1	4	1	0		0
	0			1	0	1	6 Years	1
	0					1		1
	0							1
	1	1	0	3	1	1	7 Years	1
	0	4	1			1	1 Year	0
	0	4	1	1	0	1	8 Years	1
						1	2 Years	0
	1	2	1	2	1	1		0
	0					1		0
	0	4	1	1	0	1	10 Years	0
	0					1		1
	0					1	4 Months	0
N/A		3	1	2	1	1	12 Years	1
	1	3	1	1	0	1	10 Years	1
	1	3	1	1	0	1	18 Years	1
	1	2	1	2	1	1	6 Years	0
	0					0		0
		4	1	1	0	1	10 Years	0
	0	3	1	1	0	1	5 Months	1
	1	2	1	2	1	1	3 Years	1
Was always attracked t		3	1	1	0	1	12 Years	1
		2	1	1	0	1		1
	0					1	10 Years	0
	0					1	5 Years	0
	1	1	0	3	1	1	1 Year	1
	0			2	1	1		0
	1	3	1	1	0	1	3 Years	1
	0					1		1
						1	3 Years	0
	1	2	1	2	1	1	5 Years	1
No		2	1	2	1	1	15 Years	1
	1	1	0	3	1	1	N/A	1
	0					1	6 Years	0
	0	4	1	1	0	0		0

	1	1	0	2	1	1	9 Years	0
Withdrawa	1	2	1	2	1	1	All life	1
	0			2	1	1		1
	1	2	1	1	0	1	4 Years	1
	1	1	0	2	1	1	3 Years	0
	0			1	0	1	2 Years	1
	1	1	0	4	1	1		0
	0	4	1			1	4 Years	1
Able to use	1			2	1	1		1
	1	2	1	1	0	1	3 Years	1
	0					1		1
	0					1		1
Expelled frc	0					1	20 Years	1
	0					1	2 Months	1
	1	1	0	2	1	1	10 Years	1
		2	1	1	0	1	3 Years	1
	1							
	1	1	0	2	1	1	3 Years	1
	1	2	1	1	0	1	3 Years	1
	0	4	1					
Discriminat	0					1		0
	0			2	1	1		0
	1	2	1	2	1	0		1
	1	3	1			1	1 Year	0
	0			1	0			0
	0	1	0	1	0		2 Months	0
	1	3	1	2	1	1	10 Years	1
	0					1	1 Year	0
	0					1	1 Year	0
	0							0
		4	1	1	0	1		0
	0					1	3 Years	0
	0			1	0	1	2 Months	0
	1	3	1	2	1	1	9 Months	0
		2	1	2	1	1		0
	1	2	1	2	1	1	5 Years	0
	1	3	1	2	1	1	2 Years	0
	0			1	0	1		1
	0							0
	0					1	3 Years	0
	0			1	0	1		0
	1	2	1	1	0	0		0
	1	2	1	2	1	1	N/A	0
	0			1	0	1	2 Years	0
		2	1	2	1	1		
	0	3	1	2	1	1	1 Year	0
	0			1	0	0		0
Nothing to	0	4	1	1	0	0		1
	0					1	10 Years	0
	0					1	2 Years	0
	0					0		0

No	0					0		0
	0					1	2 Years	0
	1	3	1			1		0
		4	1	1	0	1	3 Years	1
	0					1		0
	0							0
	0					1	1 Year	0
	0					1	2 Years	0
	1	1	0	2	1	0		0
	0					0		0
	1	3	1	3	1	1		0
	0	4	1			0		0
	0	4	1	1	0	1		0
	0	4	1			1	3 Year	1
	0			2	1	1	6 Years	0
	1			2	1	0		
		3	1			1	10 Years	1
	0			1	0	0		0
	0							
	1	1	0	2	1	1	4 Years	0
	0			2	1	0		0
Helped me	0					1	2 Years	1
	0					0	12 Years	1
	0			2	1	1	4 Years	0
	0							0
	0					1	2 Years	0
	1	2	1	2	1	1	6 Years	1
		3	1	2	1	1		1
		1	0	2	1	1		1
	1	1	0	2	1	1	2 Years	1
	0					1		0
	0	4	1			1	2 Years	0
	0					0		1
	1	1	0	3	1	1	5 Year	1
	0					1	18 Years	0
	0	2	1	1	0	1		0
	0					1	2 Years	0
	0					1	4 Years	0
	0			1	0	1	2 Years	0
	0							
	0					1		0
	1	2	1	1	0	1		0
About PrEP	1	2	1	1	0	1	2 Years	0
N/A	1	1	0	3	1	1		0
	0					1	1 Year	0
	1	2	1	2	1	1	5 Years	1
		2	1	1	0	1	3 Years	0
	0			1	0	1	3 Years	0
		4	1			1	6 Years	0
	1			2	1	1	5 Years	0
	0			3	1	1	2 Years	1

0			1	0	1		1
0	3	1	1	0	0		0
0	3	1	1	0	0		0
	4	1	1	0	0		0
0			1	0	0	N/A	0
	2	1	1	0	1		0
0			1	0	0		0
0					0		0
0							0

MSMMSW	Q110	Q111	If_Other	Q112	Q112rec	If_ever_ma	Q113	Q114
Identity as MSM or MSW	Identity transgender	Sexual Orientation	If Other	Marital status	Marital status	If ever married	Sexual Attraction	Sexual partners last 6 months
				1	0			
	0			1	0		3	4
								4
				1	0		2	2
	0			1	0		2	3
				3	1			
1	0	1		1	0		1	3
1	1	1		1	0		1	3
1	0	1		1	0		1	3
1	0	1		1	0	1	1	4
1	0	1		1	0		1	3
1	0	1					1	2
0	0	1		1	0		1	2
1	0	1		1	0		1	3
1	0	1		1	0	1	1	3
1	0	1		1	0		1	3
1	0	1	Sexual F	1	0		3	2
1	0	1		1	0		1	3
1	0	1		1	0		1	3
1	0	1		1	0		1	3
0	1	1		1	0		1	3
0	0	1		1	0		1	2
1	0	1		1	0		1	3
1	0	1		1	0		1	3
0		1		1	0		1	3
0	0	1		1	0		1	1
0	0	1		3	1	1	1	2
1		1		1	0		1	3
1	1	1		3	1		2	1
0	0	1		1	0		1	3
0	0	1		1	0	1	1	4
1	0	1		1	0		1	1
0	0	1		3	1	1	1	1
1	0	1		4	1	1	1	3
0	0	1		1	0	1	1	1
0	1	1		3	1		1	3
0		1		1	0		1	1
0		1		1	0	1	1	1
0	0	1		1	0		1	1
1	0	1		3	1	1	1	3
1	0	1		1	0		1	2
1	1	1		1	0	1	1	1
1	0	1		1	0		1	1

1	1	1		1	0		1	3
1	0	1		1	0		1	1
1	0	1		3	1	1	1	2
1	0	1		1	0		1	3
1		1		1	0	1	1	1
1	1	1		1	0		1	1
1	1	1		1	0		1	1
1	0	1		1	0		3	2
1	0	1		1	0		1	3
1	0	1		3	1	1	1	3
1	1	1		1	0		1	2
1	0	1		1	0		1	3
1	0	1		1	0		1	3
0	0	1		1	0		1	2
0	0	1		1	0		1	3
0	0	1		1	0		1	2
1	0	1		1	0		1	2
0	0	1		1	0		1	1
1	0	1		1	0	1	1	4
1	0	1		1	0		3	3
1	1	1		1	0		1	1
0	1	1		1	0		1	2
1	0	1		1	0			3
0	0	1		3	1	1	1	1
1	0	1		1	0		1	3
1	1	1		1	0	1	1	3
0	0	1		1	0		1	2
0	1	1		1	0		2	2
1	0	1		1	0	1	1	3
0	1	1		1	0	1	1	3
0	1	1		1	0		1	4
1	0	1		1	0		1	3
0	0	1		1	0		1	2
0		1		1	0		1	2
1	0	1		1	0	1	1	4
1	0	1		1	0	1	1	2
1	0	1		1	0		1	2
1	0	1		1	0		1	3
1	0	1		1	0		1	3
1	0	1		1	0		1	2
1	0	1		1	0		1	3
0	0	1		4	1	1	1	2
1	0	1		1	0		1	2
1	0	1		1	0		1	3
0	1	1		3	1	1	1	1
1	0	1		1	0		1	2
1	1	1		1	0	1	1	2
0	0	1		1	0		1	1
1	0	1		1	0		1	3
1	0	1		1	0		1	3
1	0	1		1	0		1	2
1	0	1		1	0		1	1

1	0	1		1	0		1	1
1	0	1		1	0		1	3
1	0	1		1	0		1	2
0	0	1		1	0		1	1
1	0	1		1	0		1	2
0	0	1		1	0		1	1
1	0	1		1	0		1	1
1	1	1		3	1	1	1	3
1	0	1		1	0		1	1
1	0	1		1	0		1	2
1	0	1		1	0		1	3
1	0	1		1	0		1	2
1	0	1		1	0		1	3
0		1		1	0		1	1
1	0	1		1	0		1	2
0	0	1		1	0		1	1
1	0	1		1	0		1	3
1	0	1		1	0	1	1	4
1	0	1		1	0		1	3
1	0	1		4	1		1	3
1	0	1		1	0	1	3	2
0	0	1		1	0		1	1
1	0	1		1	0		1	2
1	0	1		1	0		1	3
1	0	1		1	0		1	3
1	0	1		1	0		1	3
1	0	1		1	0	1	1	2
1	0	1		1	0		1	3
1	1	1		1	0		1	1
1	1	1		1	0	2	1	2
0	0	1		3	1	2	3	3
1	0	1		2	1	2	2	2
0	0	1		1	0		1	3
0		1		1	0		1	1
0	0	1		1	0		1	1
1	0	1		1	0		1	2
0	1	1		1	0	2	3	2
1	0	1		1	0		1	3
0	1	1		1	0	2	1	2
0	0	1		1	0		1	3
0	0	1		1	0		1	
1		1		1	0		3	2
0	0	1		1	0		3	3
0	0	1		1	0		3	3
1	0	1		1	0		3	3
1	0	1		1	0		1	2
1	1	1		1	0		1	1
0	1	1		1	0		1	2
1	1	1		1	0		1	4
1	0	1		1	0		1	2
1	0	1		2	1		2	3
1	0	1		1	0		1	2

0	0	1		1	0		2	4
1	0	1		1	0	2	2	3
1	1	1		1	0	1	1	3
1	0	1		1	0	1	1	1
1	0	1		1	0	1	1	3
1	0	1		1	0	1	1	3
1	0	1		1	0	1	2	2
1	0	1		1	0	2	3	1
1	0	1		1	0		1	1
0	0	1		4	1	2	4	3
1	0	1		1	0	1	1	3
1	0	1		1	0		1	2
0		1						
0		1		1	0		3	2
0		1		1	0			3
1	0	1		1	0		1	4
0	0	1		1	0		1	1
0	0	1		1	0		1	1
0		1		1	0		1	1
0	0	1		3	1	1	1	2
1	0	1		1	0		1	2
0		1		1	0		1	1
0	0	1		1	0	1	1	3
0	0	1		1	0		1	3
0	0	1		1	0		4	2
0		1		1	0			
1	0	1		1	0		1	4
0	0	1		1	0		1	2
0	0	1		1	0		3	2
0	0	1		1	0		1	3
1	0	1		1	0		1	2
1	0	1		1	0		1	3
0	0	1		1	0		1	3
1	0	1		1	0		1	3
0	0	1		1	0		2	2
0	0	1		1	0		1	1
1		1		1	0		1	2
0	1	1		1	0		2	2
1	1	1		1	0		1	1
1	0	1		1	0		1	1
0	0	1		1	0		1	2
1	1	1		3	1	1	1	2
1	1	1		2	1		1	2
0	1	1		1	0		1	2
0	0	1		1	0		3	2
1	0	1		1	0		1	3
0	0	1		4	1	2	1	4
0		1		4	1	1	1	2
0	0	1		1	0		3	2
1	1	1		1	0	1	1	2
0	0	1	N/A	1	0		1	2
1	0	1		1	0		1	3

0	0	1		1	0		1	2
1	1	1		3	1	1	1	2
0	0	1		1	0		1	3
1	0	1		1	0		1	3
1	0	1	Yes	1	0		3	2
1	0	1		1	0		1	2
1		1		1	0		1	2
1	0	1		1	0		1	3
1		1		1	0		1	2
1	0	1		1	0		1	2
1	1	1		1	0	1	1	2
0	0	1		1	0		1	1
1	0	1		1	0		1	1
0	0	1		1	0		1	2
1		1		1	0		1	3
1	1	1		1	0		3	3
0	1	1		3	1	2	2	3
1	0	1		1	0	1	1	2
1	1	1		1	0		3	3
1	1	1		1	0	1		3
1	0	1		3	1	1	1	1
0	0	1		1	0		1	4
1	0	1		1	0	1	1	3
0	0	1		1	0		1	3
1	0	1		3	1	1	1	1
0	0	1		1	0		1	1
1	0	1		1	0		1	2
1		1	N/A	1	0		1	3
0		1						
1	1	1		1	0		3	2
1	0	1		1	0		1	1
1	0	1		1	0	1	1	2
1	0	1		1	0	1	1	3
1	0	1		1	0	1	3	3
0	1	1		1	0		1	1
0	0	1		1	0		1	3
1		1		1	0		3	2
1	0	1		1	0		1	3
1	0	1		2	1		1	3
1	0	1		1	0	1	1	3
1	0	1		1	0		1	3
0	0	1		4	1		1	3
1	0	1		1	0		1	2
0	0	1		1	0		1	1
1	0	1		1	0		1	3
1	0	2		1	0		3	3
0	0	2		1	0		3	2
1	0	2		1	0		3	3
1	0	2		4	1		1	2
1	0	2		1	0		3	3
0	0	2		1	0		1	1
0	0	2		1	0		1	4

0	1	2		3	1	2	1	3
1	0	2		4	1	2	3	3
1	0	2		1	0		1	3
1	0	2		1	0		3	2
0	0	2		1	0	1	1	2
1	1	2		1	0	1	1	2
1	0	2		1	0		2	1
1	1	2		1	0		3	2
1	0	2		3	1	2	3	3
1		2		1	0		1	3
1	0	2		1	0		3	3
1	0	2		1	0		3	3
1	0	2		1	0	1	3	2
1	0	2		1	0		1	2
1	1	2		1	0		3	2
1	0	2		1	0	1	1	
0		2						
1	0	2		1	0		3	3
1	0	2		1	0		3	3
0		2						
0		2		1	0			1
0	0	2		1	0		1	2
1	0	2		4	1	2	3	3
0	0	2		1	0		3	3
1	0	2		1	0		3	1
0	0	2	Both sexual	1	0		3	1
1	0	2		4	1	2	1	3
1	0	2		1	0		3	1
0	0	2		1	0		3	1
1	0	2		1	0		4	2
0		2						
1	0	2		1	0		3	2
0	0	2		1	0		3	1
1	0	2		1	0		3	1
0	1	2		1	0		3	1
1	0	2		1	0		1	3
0	0	2		1	0		3	2
0	1	2		1	0		3	1
1	0	2		1	0		3	3
1	0	2		1	0		3	2
1	1	2		1	0		3	2
0	0	2		1	0		3	3
0	0	2		1	0	2	2	2
0	0	2		1	0		3	2
0	0	2		1	0		1	2
0	1	2		4	1		2	2
0	0	2		1	0		3	3
0	0	2		1	0		2	3
1		2		1	0		2	3
1		2		1	0		3	2
1	1	2		1	0		1	1
1	0	2		1	0		2	4

0	1	2		1	0		3	3
1	0	2		1	0		3	2
0	1	2		1	0		1	1
1	0	2		1	0		3	3
0		2		1	0			3
0	0	2		1	0		4	2
0	0	2		1	0		4	3
0	0	2		1	0		3	4
1	0	2		1	0		3	2
0	0	2		3	1	2	3	3
0	0	2		1	0		3	
0	0	2		3	1	3	3	2
0		2		3	1	3		1
0	0	2		1	0		1	3
1		2		1	0		1	3
0	0	2		1	0	1	3	2
0		2		1	0			
1		2		3	1		1	1
0	1	2		1	0		4	3
0		2						2
0	0	2		3	1	2	3	1
1	0	2		1	0		3	2
1		2		1	0		3	1
1	0	2		1	0		1	3
0	1	2		1	0		3	3
0	0	2		1	0		3	
1	0	2		4	1		3	3
1	0	2		1	0		3	3
1	0	2		3	1		3	2
1		2		3	1	2	1	3
1	0	2		1	0		1	3
	0	4		1	0		3	1
0	0	2		1	0		3	3
1	1	2		1	0		1	2
	0	3		3	1	1	1	1
0	1	2					1	3
1	0	2		2	1	2	2	2
0	1	2					1	3
0		2		1	0		3	3
		4		1	0		3	1
0		2		1	0		3	1
0	0	2		1	0		3	2
0	0	2		1	0		3	3
0	0	2		1	0	2	3	2
0	0	2	N/A	1	0		3	2
0	0	2		1	0		3	4
1	0	2		1	0		3	3
0	0	2		1	0		3	2
0		2		1	0		3	1
0	0	2		1	0		3	3
0	0	2		3	1	2	3	2
	1	3		4	1		3	2

1	3		1	0	1	1	1
0	3		1	0		4	2
1	3		1	0		4	2
0	3		1	0	2	2	3
0	3		1	0		2	3
0	4		1	0	1	1	4
0	4		1	0		3	3
0	4		1	0		4	1
0	4		1	0	2	2	3

Q114Rec	Q115	Q115If_yes	Q115If_yes	Q116	Q117	Q117Ifyes	Q118	Q119
Sexual partners last 6 months	Use condom during sex	If yes how often	If yes how often do you use condom	Prefer top or bottom	Are you currently using	If to hard drugs which ones	Had anal sex after alcohol/hard drug	Do you use condom during
				2	1		0	1
1	1	2	1	3	1		0	2
1	1	1	0					
1	1	1	0		2	Shash/Kate	0	4
1		3	1	2	1		0	
				3	1			1
1	1	2	1	2	1		1	2
1	1	1	0	2	1		1	1
1	1	1	0	1	1		0	
1	1	1	0	1	1		1	1
1	1	2	1	3	1		1	3
1	1	3	1	3	1		1	2
1	1	1	0	1	1		1	1
1	1	2	1	2	2		0	1
1	1	1	0	1	1		1	1
1	1	3	1	3	1		1	2
1	1	1	0	3	1		1	1
1	1	3	1	1	1			4
1	1	2	1	2	2	Bhang	1	2
1	1	1	0	1		No	0	4
1	1	3	1	2	4		0	3
1	1	1	0	1	1	Beer	0	1
1	1	1	0	3	1		0	1
1	1	1	0	1	1		1	1
1	1	3	1	1	1		1	3
0	1	1	0	1	1	Beer	1	1
1	1	2	1	1	4		0	1
1	1	3	1	1	1		0	2
0	1	1	0	1	1		1	1
1	0			1	1		1	4
1	1	1	0	3	1		0	1
0	1	1	0	1			0	1
0	1	1	0	3	1		1	1
1	0	4	1	3	1	Ndovu, kub	1	4
0	1	3	1	3	1		1	1
1	0			1	2		1	1
0	1	1	0	1	1		1	1
0	1	3	1	2				
0	1	1	0	1	1		0	1
1	1	2	1	3	1		1	3
1	1	2	1	3	1		1	2
0	1	2	1	1	2		0	2
0	1	2	1	3	1		1	2

1	1	1	0	1	1		1	1
0	1	3	1	1			0	1
1	1	1	0	3	1		1	1
1	1	2	1	3	1		1	3
0	1	1	0	1	1		1	1
0	1	1	0	1			0	1
0	1	1	0	1			0	4
1	1	3	1	1	1		0	3
1	1	1	0	3	1		1	1
1	1	2	1	3	1		1	2
1	1	1	0	3	1		1	1
1	1	2	1	3	1		1	3
1	1	3	1	3	1		1	3
1	1	1	0	3	1		1	2
1	1	1	0	1	2	Bhang	1	3
1	1	1	0	2	1		1	1
1	1	1	0	1	1		0	3
0	1	1	0	1	1		0	
1	1	1	0	1	1		1	1
1	1	3	1	3			0	2
0	1	1	0	1	1		1	1
1	1	2	1	3			0	
1	0			2	3		1	4
0	0			2	1		0	4
1	1	2	1	1	1		1	2
1	1	3	1	3	2	Cocaine	1	3
1	1	3	1	1			1	3
1	1	2	1	1	2		0	2
1	1	3	1	2			1	4
1	1	3	1	2	1		1	3
1	1	1	0	3			1	1
1	1	3	1	3	1		1	3
1	1	1	0	1	1		1	2
1	1	2	1	1	1		0	1
1	1	1	0	1	1		1	1
1	1	1	0	3	1		1	1
1	1	2	1	3			0	
1	1	1	0	2	1		1	1
1	0			3			0	4
1	1	1	0	1	1		0	1
1	1	1	0	2	1		1	1
1	1	3	1	3				
1	1	1	0	2	1		1	1
1	0			3			0	4
0	0			2	1		1	4
1	1	1	0	2	1		1	1
1	1	2	1	2	1		1	2
0	0			1			0	
1	1	2	1	1	1		0	2
1	1	1	0	2	1		1	1
1	1	1	0	2	1		1	1
0	0			1			0	

0	1	1	0	2	1		1	1
1	1	2	1	2	1		0	3
1	0			3			0	4
0	1	1	0	1	1		0	1
1	1	1	0	2	1		1	1
0	0			1			0	
0	1	1	0	2	1		1	1
1	1	3	1	2	1		1	3
0	1	1	0	2	1		1	1
1	1	1	0	3	1		0	1
1	0			3			0	4
1	1	1	0	2	1		1	1
1	0			1	1		0	4
0	0			1			0	
1	1	1	0	1	1		1	1
0	0			1			0	
1	1	1	0	2	1		1	1
1	1	1	0	2	1		1	1
1	1	1	0	2	1		1	1
1	1	1	0	2	1		1	1
1	1	1	0	2	1		0	1
0	1	1	0	3	1		1	1
1	1	1	0	2	1		1	1
1	1	1	0	3			0	4
1	0			3			0	4
1	1	2	1	3	1		1	3
1	1	2	1	1	1		1	2
1	0			3			0	4
0	1	1	0	3	1		0	1
1	1	1	0	1	1		1	1
1	1	1	0	1	1		1	1
1	1	2	1	2	2		0	2
1	1			1			0	1
0	1	1	0	2		No	1	3
0	1	1	0	1				
1	1	3	1	3			0	
1	1	2	1	3	2		0	4
1	1	1	0	1			0	
1	1	1	0	1	1		1	1
1	1	1	0	3	1		0	1
1	1	1	0	3				
1	1	1	0	3	5		1	1
1	1	1	0	3	2	Bhang	1	1
1	1	1	0	3	2	Bhang	1	1
1	1	3	1	3	1		1	3
0	1	1	0	3	1		1	1
1	1	1	0	2		No		
1	1	1	0	1	2	Marijuana	1	1
1	1	1	0	1	2	Bhang	0	1
1	1	1	0	2			0	1
1	1	1	0	1			0	1

1	1	1	0				0	
1	1	1	0	3	1		0	3
1	1	2	1	2	1		1	1
0	1	2	1	3	1		0	3
1	1	2	1	1	1		1	2
1	1	2	1	1	1		0	1
1	1	2	1	1	1		1	2
0	1	1	0	3	1		1	1
0	1	2	1	1	1		1	2
1	1	1	0	3	1		0	1
1	1	1	0	3	1		1	1
1	1	1	0	3				1
1	1	2	1	1	1		1	2
1	1	2	1		1		0	
1	1	1	0	3	1		0	1
0	1	3	1	2	2	Bhang	1	4
0	1	1	0	2	2	Cock/Bhan	1	4
0	1	1	0	1	1		0	1
1	1	2	1	1	1		1	2
1	1	1	0	1	2		1	1
0	1	1	0	3			0	1
1	1	2	1	3	1		1	1
1	1	2	1	2	1		1	2
1	0						0	
	1	2	1		1		1	2
1	0			3	1		0	4
1	1	1	0	1	1		1	3
1	1	2	1	3			0	2
1	1	1	0	3	1		0	1
1	1	3	1	2			1	
1	1	1	0	3			0	1
1	1	1	0	1	1		1	2
1		3	1	1				
1	0	4	1	3	1	Bottles	0	4
0	1	1	0	3			1	1
1	1	1	0	3			0	1
1	1	1	0	3	2	Weed	0	1
0	1	1	0	2			0	1
0	1	1	0	2			0	
1	1	1	0	1		N/A	0	1
1	1	2	1	2	1		1	1
1	1	1	0	1	1		1	1
1	1	2	1	3	1		0	3
1	1	2	1	3	1	Beer	0	2
1	1	1	0	2				1
1	1	1	0	1	5		0	
1	1	1	0	2	1		1	3
1	1	1	0	2	1	Beer	0	
1	1	1	0	3	2		0	4
1	1	2	1	1	1		1	1
1	1	2	1	1			1	2

1	1	3	1	2	1		1	3
1	1	2	1	3	1		1	2
1	1	1	0	3	1		1	1
1	1	1	0	2			0	4
1	1	1	0	2	2	Miraa	1	3
1	1	3	1	3	1		1	3
1	1	2	1	1	1		1	2
1	0			2			0	
1	1	2	1	1	1		1	3
1	1	1	0	1	1		1	1
1	1	1	0	1		No	0	4
0	1	1	0	2	1		1	1
0	1	1	0	1	1		1	1
1	1	3	1	3	1		1	2
1	1	3	1	1	1		1	3
1	1	1	0	3	2		1	1
1	1	3	1	3	1		1	3
1	1	1	0	2			0	1
1	1	2	1	3	1		0	3
1	1	2	1	2	4		0	1
0	1	1	0	3	1		1	1
1	1	1	0	2			0	1
1	1	3	1	3	2	Cigar	1	2
1	1	1	0	1	1		1	1
0	1	1	0	1			0	4
0	1	2	1	2			0	2
1	1	1	0	1	2	Cocaine	0	1
1	1	2	1	2		N/A	1	2
1	1	2	1	1			0	
0	1	3	1	3				
1	1	1	0	3	1		1	1
1	1	1	0	3	1		1	1
1	1	1	0	3		Nop	0	1
0	1	1	0	3			0	
1	0			3	1		1	4
1	1	1	0	3			0	1
1	1	2	1	3	1		1	2
1	1	3	1	2	1	Tusker	1	3
1	1	3	1	3			1	3
1	1	1	0	3			0	
1	1	1	0	1	1		1	1
1	1	1	0	3	1		1	1
0	1	2	1	2	1		1	2
1	1	1	0	3	1		1	1
1	1	2	1	3	1		1	3
1	1	2	1	3	3	Bhang and	1	3
1	1	1	0	1	1		0	1
1	1	1	0	3	1		1	1
1	1	1	0	3	2	Bhang	1	1
0	1	1	0	1	4			
1	1	1	0	1	1		1	1

1	1	1	0	3	1		1	1
1	1	2	1	3	1		1	2
1	1	2	1	3	1		0	2
1	1	3	1	1			1	3
1	1	1	0	1	1		1	4
1	1	1	0	2	1		1	1
0	1	3	1	1	1	Whysky	1	1
1	1	1	0	1	1		0	1
1	1	2	1	3	1		1	2
1	1	1	0	2	1		1	1
1	1	1	0		1		0	1
1	1	1	0	1	1		1	2
1	1	2	1	3	1		1	2
1	1	1	0	1	1		0	1
1	1	1	0	3	1	Beer	1	1
	1	1	0	1	1		0	1
				3	1		1	3
1	1	2	1	1	1		1	1
1	1	2	1	1	1		1	2
				2	1		1	1
0	1	1	0	3		No		
1	1	1	0	1	1		1	1
1	1	3	1	1	1		0	
1	1	2	1	3	1		1	3
0	0	3	1	1	2	Out/Muua	0	1
0	0	3	1	1	2	Out/Muua	0	1
1	1	2	1	3	1		0	
0	1	1	0	3			0	1
0	1	1	0	1	1		0	4
1	1	2	1	1				1
1	1	1	0	1			0	
0	1	3	1	3	1		1	3
0	1	2	1	3			0	2
0	1	1	0	3			1	1
1	1	1	0	3			0	1
1	1	1	0	1				
0	1	1	0	3			0	1
1	1	1	0	1			0	1
1	1	1	0	3	1		1	1
1	0			3	1		1	3
1	1	1	0	3	1		0	1
1	1	1	0	1	2	Herb	1	2
1	1	1	0	3	1		1	1
1	1	1	0	1	1		1	1
1		2	1	1	2		0	
1	1	2	1	1				
1	1	2	1	3	1		0	
1	0	4	1	3	1		1	4
1	1	3	1	1	1		1	3
0	1	3	1	2	1		1	2
1	1	1	0	3	1		0	4

1	1	2	1	3	1		1	1
1	0			3	1		1	4
0	1	2	1	3	1		1	1
1	1	2	1	3			0	
1	1	2	1	3			0	1
1	1	1	0	2	1		0	1
1	1	3	1		1		1	
1	1	1	0	1	1		0	1
1	1	2	1	2	1		1	2
1	1	2	1	1	1		1	3
	1	1	0	3			1	1
1	1	2	1	3	1		1	2
0	1	2	1	1	1	Alcohol	0	
1	1	3	1	3	3	Mandrax	1	3
1	1	1	0	2	1		0	1
1	1	1	0	3	1		0	1
					1	Jemson		
0	0			1	1		1	4
1	1	3	1	3	1		1	3
1	1			1			0	1
0	1	2	1	1	1	Beer	1	2
1	1	1	0	1			0	
0	0			1	1		0	3
1	1	1	0	2		No	0	4
1	1	2	1	1			0	4
	1	1	0				0	
1	0			1	1		1	3
1	1	2	1	3	1		1	2
1	1	1	0	3				
1	1	1	0	1			0	1
1	1	2	1	3	1		1	2
0	1	1	0	3	1		1	2
1	1	1	0	2	1		1	1
1	1	1	0	1	1		0	2
0	1	3	1	1	1		0	4
1	1	2	1	3	1		1	3
1	1	2	1	3	1		0	2
1	1	2	1	3	1		1	3
1	1	1	0	1			0	1
0	1			3	1		0	1
0	1	1	0	1	1		0	
1	1	1	0	3	1		0	4
1	1	1	0	1			0	
1	1	1	0	3	1		1	1
1	1	1	0	1			0	1
1	1	1	0	3	1		0	
1	1	2	1	1	1		1	2
1	1	2	1	3				
0	1	1	0	3	1		1	1
1	1	1	0	1			0	1
1	1	1	0	1	1		1	1
1	1	2	1	1	2	Hard drugs	1	2

0	1			2	1		1	2
1	1	2	1	2	1		0	4
1	1	2	1	1	2	Bhang	0	3
1	1	1	0	3	1		0	
1	1	1	0					
1	1	2	1	2	2	Bhang	0	1
1	1	1	0	3	1		0	4
0				1			0	4
1	1	1	0		3	Bhang	0	

Q119Rec	Q120	Q120If_yes	Q120If_yes	Q120If_oth	Q121	Q121If_yes	Q122	Q123HIV
Do you use condom during	Did you use lubricant in last	If yes	Type of lubricant in last sexual act	If other specify	Have you ever had any mental	If yes explain	Insurance cover	HIV/AIDS
0	1	4	1.00		0		0	7
1	1	4	1.00		0		0	7
1	0				0		0	
	0				0		0	
0	1	1	0.00					
1	1	1	0.00		0		1	7
0	1	1	0.00		0		1	1
	1	1	0.00		0		1	6
0	1	1	0.00		0		0	1
1	1	1	0.00		0		1	2
1	1	1	0.00		0		0	2
0	1				0		0	8
0	1	1	0.00		0		0	
0	1	1	0.00		0		0	6
1	1	1	0.00		0		0	6
0	1	1	0.00		0		1	5
1	0				0		0	6
1	1	2	1.00		0		0	6
1	1	1	0.00		0		1	5
1	1	2	1.00		0		1	6
0	1	1	0.00		0		0	6
0	1	2	1.00		0		0	7
0	1	1	0.00		0		1	5
1	1	1	0.00		0			
0	1				0		0	
0	1	1	0.00		0		1	7
1	1	1	0.00		0			6
0	1	1	0.00		1		1	
1	1	4	1.00		0		1	5
0	1	1	0.00		0		1	5
0	1	2	1.00		0			5
0	1	1	0.00		0		1	6
1	1	5	1.00		1	Mental	1	6
0	1	1	0.00		0		1	5
0	0	3	1.00		0		1	6
0	1	2	1.00		0		0	5
	0				0		0	6
0	1	1	0.00		0		1	
1	1	1	0.00		0		1	6
1	1	4	1.00		0		0	5
1	0	2	1.00		1		1	1
1	1	1	0.00		0		0	6

0	1	1	0.00	0		1	6
0	1	2	1.00	0		0	6
0	1	1	0.00	0		1	6
1	1	1	0.00	0		0	6
0	1	1	0.00	1		0	5
0	0			0		0	
1	0			0		0	6
1	1	4	1.00	0		0	5
0	1	1	0.00	0		0	6
1	1	1	0.00	0		0	6
0	1			0		0	6
1	1	1	0.00	0		0	7
1	1	1	0.00	0		0	7
1	1	1	0.00	0		0	5
1	1	1	0.00	0			6
0	1	1	0.00	0		0	
1	1	1	0.00	1		0	6
	1			0		0	6
0	1	1	0.00	0		0	6
1	1	1	0.00	0		0	5
0	1	1	0.00	0		1	6
	1	3	1.00	0		0	6
1	0			1	Depression	0	6
1	1	1	0.00	0		1	6
1	1	1	0.00	0		1	6
1	0	4	1.00	1	Depression	0	6
1	1	1	0.00	0		0	6
1	1	1	0.00	1		1	5
1	1	1	0.00	0		0	5
1	1	1	0.00	0		0	8
0	1	1	0.00	0		0	7
1	1	1	0.00	0		0	8
1	1	1	0.00	0		0	6
0	1	2	1.00	0		0	6
0	1	1	0.00	0		0	6
0	1	1	0.00	0		0	6
	1	1	0.00	0		0	6
0	1	1	0.00	0		0	8
1	1	1	0.00	0		0	5
0	1	1	0.00	0		0	5
0	1	1	0.00	0		0	8
	1	1	0.00	1	Depression	0	7
0	1	3	1.00	0		0	8
1	1	4	1.00	0		0	5
1	1	1	0.00	0		1	5
0	1	3	1.00	0		0	8
1	1	1	0.00	0		1	5
	0			0		0	
1	1	1	0.00	0		0	
0	1	1	0.00	0		0	8
0	1	3	1.00	0		0	8
	0			0		0	

0	1	3	1.00	0		0	8
1	1	4	1.00	0		0	6
1	1	1	0.00	0		0	5
0	1	1	0.00	0		1	5
0	1	2	1.00	0		0	6
	0			0		0	
0	1	1	0.00	0		0	6
1	1	1	0.00	0		0	6
0	1	4	1.00	0		0	8
0	1	4	1.00	0		0	5
1	1	3	1.00	0		0	5
0		2	1.00	0		0	8
1	1	1	0.00	0		0	8
	0			0		0	
0	1	1	0.00	0		1	6
	0			0		0	
0	1	3	1.00	0		0	6
0	1	1	0.00	0		0	6
0	1	4	1.00	0		1	8
0	1	1	0.00	0		0	6
0	1	2	1.00	1		0	5
0	1	1	0.00	0		1	6
0	1	3	1.00	0		0	8
1	1	4	1.00	0		0	6
1	1	2	1.00	0		0	5
1	1	1	0.00	0		0	
1	1	1	0.00	0		0	6
1	1	2	1.00	0		0	
0	1	3	1.00	0		1	8
0	1						
0	1	4	1.00				5
1	0	2	1.00	0		1	
0	1	1	0.00	0		0	6
1	1	2	1.00	0		0	5
	1	1	0.00	0		0	6
	1	4	1.00	0		0	
1	1	4	1.00	1		0	
	1	1	0.00	1	Feeling con	0	
0	1	1	0.00	1		1	
0	1	1	0.00	0		1	
	0			0		1	5
	1	2	1.00	0			5
0	0			0		0	
0	0			0		0	5
0	0			0		0	5
1	1	1	0.00	0		0	6
0	1	4	1.00	0		0	6
	1	1	0.00	0		0	
0	1	4	1.00	0		0	6
0	0			0		0	6
0	1	1	0.00	0			8
0	1	2	1.00	0			6

							0	6
1	0	1	0.00		0		1	6
0	0	3	1.00		1			
1		2	1.00		0		0	6
1	1	3	1.00		0		0	6
0	1	1	0.00		0		0	6
1	1	3	1.00		0		0	6
0	1	4	1.00		0		0	5
1	1	3	1.00		1		0	5
0	0	4	1.00		0		0	5
0	1	1	0.00		1		0	5
0	1	1	0.00		0		0	
1	1				1		0	5
							0	6
0	1	1	0.00		0		0	6
1	0	2	1.00		0		1	6
1	1	5	1.00	Cooking oil	0		1	6
0	1	2	1.00		0			
1	1	2	1.00		0		0	6
0	1	2	1.00		0		0	6
0	1	1	0.00		0		0	6
0	1	1	0.00		0		0	6
1	1	1	0.00		1	Depression	0	6
							0	6
1	1							
1	1	4	1.00		0		0	5
1	1	4	1.00		0		1	6
1	1	1	0.00		0		0	6
0	1	2	1.00		0		0	5
		4	1.00		0		0	
0	1	1	0.00		0		0	6
1	1	1	0.00		0		0	6
	1	1	0.00		0		1	
1	0				0		0	6
0	1	1	0.00		0		1	
0	1	1	0.00				1	5
0	1	1	0.00		0		0	
0	1	2	1.00		0		0	6
	1	1	0.00		0		0	6
0	1	1	0.00		0		0	6
0	1	1	0.00		0		0	8
0	1	1	0.00		0	No	0	8
1	1	1	0.00		0		0	6
1	0				0		1	5
0	1	1	0.00		0		0	5
	1	4	1.00		0		0	5
1	1	1	0.00		0		0	6
	1	1	0.00		0		0	6
1	1	1	0.00		0		0	
0	1	1	0.00		0		1	7
1	1	1	0.00		0		1	6

1	1	1	0.00		0	0	6
1	0	1	0.00		0	0	
0	1	1	0.00		0	0	6
1	1	1	0.00		0	0	6
1	0				0	1	
1	1	1	0.00		0	0	6
1	1	3	1.00		0	0	6
	1	1	0.00		0	0	6
1	1	2	1.00		0	1	6
0	1				0	0	6
1	1	4	1.00	No	0	0	
0	1	1	0.00		0	1	6
0	1	1	0.00		0	1	6
1	1	2	1.00		0		5
1	1	1	0.00		0	1	6
0	1	3	1.00		1	1	6
1	1	3	1.00		1	0	6
0	1	4	1.00		0	0	
1	0				0	1	8
0	0				0	0	
0	1	1	0.00		0	1	6
0	1	1	0.00		0	1	6
1	1	1	0.00		0	0	6
0	1	1	0.00		0	0	5
1	1	1	0.00		0	1	6
1	1	1	0.00		0	1	6
0	1	1	0.00		0	0	
1	1	1	0.00		0	0	
	1	4	1.00		0	0	7
	1	1	0.00		0	0	
0	1	1	0.00		0	1	5
0	1	1	0.00		0	1	7
0	1	1	0.00		0	0	
					0	1	5
1	1	1	0.00		0	0	6
0	1				0	0	
1	1	1	0.00		0	0	6
1	1	1	0.00		0	0	
1	1	1	0.00		0	0	6
	1				0	0	
0	1	1	0.00		0	1	6
0	1	1	0.00		1	0	
1	1	1	0.00		0	0	5
0	1	2	1.00		0	1	
1	1	1	0.00		1	0	7
1	1	1	0.00		0	0	6
0	1	2	1.00		0	0	6
0	1	1	0.00		0	1	5
0	1	1	0.00		0	1	6
	1	3	1.00		0	0	
0	1	4	1.00		1	0	5

0	1	1	0.00	0		1	6
1	1	3	1.00	1	Financial c	1	8
1	1	1	0.00	0		0	5
1	1	1	0.00	0		0	5
1	1	1	0.00	1		0	6
0	1	1	0.00	0		0	6
0	1	3	1.00	0		1	
0	1	1	0.00	0		0	5
1	1	1	0.00	0		0	6
0	1	1	0.00	1	Depression	0	6
0	1	2	1.00	0		0	6
1	0			1	Had an acci	0	5
1	1	1	0.00	1	Memory Lo	0	8
0	1	1	0.00	0		0	5
0	1	1	0.00	0		1	6
0	0	2	1.00	0		0	
1	1	1	0.00	0		0	5
0	1	1	0.00	1	Bipolar	0	6
1	1	1	0.00	0		0	6
0	1	1	0.00	0		0	5
	1	1	0.00	0		0	6
0	1	1	0.00	0		0	6
	1	4	1.00	0		0	6
1	1	1	0.00	0		0	
0	1	1	0.00	1	Nil	0	6
0	1	1	0.00	0		0	6
	1	1	0.00	0		1	5
0	1	1	0.00	0		0	6
1	1	1	0.00	0		0	6
0	1	2	1.00	0		0	6
	1	2	1.00	0		0	6
1	1	1	0.00	0		1	6
1	1	4	1.00	0		0	
0	1	4	1.00	0		1	6
0	1	4	1.00	0		0	5
	1	1	0.00	0		0	6
0	1	4	1.00	0		1	6
0	0			0		0	6
0	1			0		0	5
1	0			0		1	
0	1	1	0.00	1	Ulcers	0	6
1	1	1	0.00	0		1	6
0	1			0		0	6
0	1	1	0.00	0		0	6
	1	1	0.00	0		1	5
	1	1	0.00	0		0	6
	0			0		0	5
1	0	2	1.00	0		1	5
1	1	1	0.00	0		0	6
1	1	2	1.00	0		0	5
1	0			0		0	

0	1	1	0.00	0	0	
1	1	2	1.00	0	0	6
0	1	2	1.00	1	1	8
	0			0	0	6
0	1					
0	1	1	0.00	0	0	6
	1	4	1.00	0	1	
0	1	1	0.00	0	1	6
1	1	1	0.00	0	1	6
1	1	2	1.00	0	0	5
0	1	1	0.00	0	0	5
1	1	1	0.00	0	0	6
		1	0.00	0	0	6
1	0	4	1.00	1	0	6
0	1	1	0.00	0	0	6
0	1	1	0.00	0	0	
1	0	4	1.00	0	0	6
1	1	1	0.00	0	0	6
0	1	4	1.00			
1	1	4	1.00	0	1	5
	1	4	1.00	0		
1	1	1	0.00	0	0	6
1	1	1	0.00	0	0	6
1	1	1	0.00	0	0	6
	0			0	0	
1	0	1	0.00	0	1	6
1	1	1	0.00	0	0	6
	1	1	0.00	0	1	5
0	1	1	0.00	0	1	6
1	1	1	0.00	0	0	6
1	1	1	0.00	0	0	6
0	1	6	1.00	0	1	6
1	1	1	0.00	0	0	
1	1	1	0.00	0	1	6
1	0					6
1	0	2	1.00	1	1	8
1	0					6
0	1	4	1.00	0	0	6
0	1	4	1.00	0	0	6
	1	2	1.00	0	0	
1	1	1	0.00	0	0	6
	1	2	1.00		0	6
0	0			0	0	6
0	1	1	0.00	0	1	6
	1	1	0.00	0	0	6
1	1	4	1.00	0	1	6
	1	2	1.00	0		6
0	1			0	0	6
0	1	4	1.00	0	0	6
0	1	1	0.00	0		6
1	1	2	1.00	0	1	

1	1	4	1.00	1	1	5
1	1	3	1.00	0	0	5
1	0	3	1.00	0	0	5
	1	1	0.00	0	0	
				0	0	6
0	1	2	1.00	0	0	
1	0	4	1.00	0	0	5
1	0			0	0	6
				0	0	6

Q123TB Tuberculo sis	Q123HCV Hepatitis C	Q123STI STI	Q123HBV Hepatitis B	Q123b Currently enrolled in any HIV care and	Q123bIf_ye If yes specify	Q124 What social pages do you	Uptake_of_	Q162 Ever heard of immediat e test and
7	7	7	7	1	Githunguri	2		1
7	7	7	7	1		1		1
						34		
				1		1		1
						1		
				1	MPEG	1		1
				1		2		1
1	2	2	1	1	I am on AR	1		1
1	1	1	1	0		1		
6	6	6	6	1	PrEP	11		0
1	1	1	1	1	Hoymas	12		1
				0		14		1
1	2	2	1	1		1		
8	8	8	8	0		11		0
				1		15		
5	5	5	5	0		10		0
5	5	5	5	1		1		1
5	5	5	5	1		1		1
	5	5				12		0
		6	5	1		20		1
5	5	5	5	0		1		1
6	5	5	5	0		21		1
				1		1		0
5	5	5	5	0		22		1
5	5	5	5			4		0
				1	PrEP Progra	2		0
				0		22		0
	5	7	7	0		11		1
5	6	6	5	0		23		1
				1		1		1
5	5	6	6	1	PrEP	12		0
5	5	5	5	0		22		0
5	5	5	5	1	PrEP	1		1
6	6	6	6	0		30		1
5	5	5	5	1	PrEP	31		1
5	5	5	5	1	PrEP	1		1
				0		1		
5	5	5	5	1		14		
5	5	5	5	1		10		
				1		32		1
6	6	6	6	0		13		0
5	5	5	5	0		1		1
		1		1		13		1
5	5	6	6	0		4		1

6	6	6	6	1		1	1
6	6	6	6	1		1	1
6	6	6	6	0		30	1
		6		1		2	1
				0		2	1
			5	0		17	0
				1		1	0
				0		14	0
				1	PrEP	2	0
6	6	6	6	1	PrEP	3	1
		6		1		2	1
6	6	7	5	1	ART	1	1
5	5	7	5	1	ART	5	1
				1		17	1
6	6	8	8	1	PrEP	1	0
7				1		1	1
6				0		1	1
				0		26	1
6	6	6	6	1	Support gr	27	1
5	6	5	5	1	Condoms a	1	1
6	6	6	6	0		1	1
				1	PrEP	32	0
				0		31	0
		6	6	1	Support gr	17	1
6	6	6	6	1		10	1
5	5	6	5	1	PrEP	11	0
	6		6	1	PrEP	2	0
			5	1		5	1
				1		5	0
8	8	8	8	1		1	1
				1		1	1
5	5	6	5	1		1	0
5	6	6	6	1		22	1
5	5	6	5	0		2	0
6	6	6	6	1		17	1
		6		1	PrEP	22	1
		6		0		1	1
6	6	6	6	1		1	1
5	5	5	5	0		1	0
6	6	5	6	0		1	1
				1		3	1
				1		12	1
				1		1	1
		5		0		2	0
5	5	5	5	1		17	0
				1		4	1
5	5	5	5	1	PrEP	4	1
				0		1	0
				0		12	0
6	6	6	6	1	On ARV	1	1
				1	ART	6	1
				0		1	0

				1		5	1
				1	PrEP	29	1
5	5	5	5	0		2	0
		6				17	1
				1		3	1
				0		2	0
				1		1	1
6	6	6	6	0		4	1
		8		1		1	1
5	5	5	5	0		17	1
				0		1	0
				1		6	1
				0		14	1
				0		1	0
		6		1	PrEP	17	0
				0		1	0
				1		4	1
6	6	6	6	0		12	1
				1		4	1
6	6	6	6	1		1	1
				1		1	1
		6		1	PrEP	14	0
				1		7	1
5	5	5	5	0		11	1
5	5	5	5	0		2	0
				1		33	1
6	6	6	6	1		1	1
		7		0		1	0
				0		1	1
5		5	5			10	
6	6	6	6	1		1	
				1	MPEG	13	1
5		5		1	Saw Hose Thika		1
						2	0
						1	0
				1		2	
				1	Githurai La	1	1
				1		1	0
				1	PrEP	1	0
				1	MPEG	1	0
5	5	5	5			1	
				0		1	0
				0		1	1
5	5	5	5	0		10	1
				1	PrEP	2	0
5	5	5	5	0		1	0
				0		1	0
6	6	6	6	0		1	1
6	6	6	6	0		2	0
				0			0
				1		13	1

6	6	6	6	0		2	
		5		1		2	
				0		34	
		6		0		3	0
				0		3	1
6	6	6	6			34	0
				1	PrEP	3	0
5	5	5	5	0		1	0
				1		3	
						10	
		5		1		2	1
						2	1
5	5	5	5	0		1	
						3	1
6	6		6	1	PrEP	1	1
5	5	6	6	1		4	
				1	MPEG	3	0
				0		5	0
6	6	6	6	0		26	1
6	6	6	6	1	Facebook	5	0
6	6	5	6	0		1	1
				0		21	1
		6		1		17	1
		6	6	0		35	0
				0		2	1
5	5	5	5	1	MPEG	3	0
				1		1	
				0		1	0
						1	0
6		6	6	1		45	1
		6	6	1	PrEP	46	
				1		1	
				0		1	1
				1	PrEP	14	
						22	
				0		1	0
6	6	6	6	0		9	0
6	6	6	6	0		1	1
5	5	5	5	0		10	0
6		6		1	Test	42	1
8	8	8	8	1	Ruiru	2	1
6	6	6	6	0		1	1
				0		10	0
				1		48	0
5	5	5	5	0		1	1
6	6	6	6	0		47	0
				1		10	1
				1		26	0
5	5	5	5	1	LVCT	15	1
				1	LVCT Healt	13	1

		6		0		2	0
				0		37	0
				0		21	0
6	6	6	6	0		1	1
				0		2	1
				1	PrEP	1	1
5		5	5	1	Use condor	17	1
				0		1	
				0		1	1
5	5	6	6	1	HIV	2	1
				1	Treat our b	2	1
6				0		50	
6	6	6	6	0		3	0
				0		1	
			6	0		1	
				1		1	1
				1		1	1
				1		1	1
				1		2	
				1	LVCT	49	1
6	6	6	6	0		1	
		6	6	0		1	0
5	5	5	5	0		2	1
6	6	6	6	1	MPEG	2	1
		6	6	0		10	
				0		4	0
				1		14	
5	5	5	5	0		2	1
				1		1	
5	5	5	5	1	On Care	38	1
6	6	6	5	1		39	1
				1	LVCT	32	1
5	5	5	5	0		10	0
6	6	6	6	1	PrEP	26	1
				0		1	1
		6	6	1		14	1
				1	LVCT	14	1
						12	1
						3	1
6	6	6	6	1	PrEP	41	
						17	0
5	5	5	5	0		1	1
						31	1
5	5	5	5	1	LVCT	1	1
2	1	6	1	1	PrEP	10	0
5	5		6			4	1
			6	1	Hoymas	16	1
6	6	6	6	1		30	1
				1	HIV testing		0
				1	Condom us	2	1

				0		1	1
6	6	6	6	1	ART	28	1
		5		1	ART	29	1
5	5	5	5	0		1	1
6		6		0		3	1
6	6	6	6	1		1	1
				0		1	0
5	5	5	5	1		1	1
5	5	5	5	1	PrEP	1	0
6	6	6	6	0		12	1
		8		0		13	0
		5		0		13	0
6		6		1	ART	31	1
5	5	5	5	1		1	1
		6		1	LVCT	30	1
				0		2	1
5	5	5	5	0		24	1
				0		1	1
5	6	6	6	0		28	1
5	5	5		1		5	
6	6	6	6	0		11	1
5	5	6	5	0		1	0
5	5	6	6	1	PrEP	1	1
				0		1	
	6	5	6	1			1
5	6	5	6	1	Protection		1
7	7	5	7	1	ART	10	
5	5	5	5	0		12	0
6	6	6	6	0		17	
		6	6	0		17	1
			5			2	1
				0			1
				0		1	0
6	6	6	6	0		3	1
		5		0		1	0
6	6	6	6	0		2	1
6	6	6	6	0		1	1
6	6	6	6	0		2	1
5	5	5	5	0		2	0
							0
7	7	7	7	0		2	0
				1	MPEG	1	1
6	6	6	6	0		14	0
	6	6		0		36	0
5	5	5	5				
						3	
5	5	5	5	0		2	0
5	5	5	5	0		1	
				0		1	0
5	5	5	5	1		1	0
				0		10	0

				1		17	1
6				0		2	0
				1		1	
				0		10	0
5		5		0		20	0
				0		11	0
5				0		2	0
	6	6	6	0		43	1
5				0		44	0
5		5		1	LVCT	17	1
				0		1	
6	6	6	6	1		2	
6				0		1	1
6	6	6	6	0		2	1
						1	0
		6		0		1	0
5	5	5	5	0		1	
				1		1	
5	5	5	5	0		1	
				1		1	
6				1		14	1
6	6	6	6	0		1	1
				0		1	1
				0		34	1
6	5	5		0		1	0
				1	MPEG	12	1
				1	SWOP	4	1
				0		4	0
		6		1	PrEP	4	0
						17	
				0		10	0
				0		1	1
				0		49	0
				0		1	1
				1		1	1
				0		1	
8	5	6	5			10	1
6	6	6	6	0		1	1
				0		3	0
		6		0		14	0
5	5	5	5	0		22	1
5	5	5	5	1	MPEG	1	1
				0		17	0
		6				10	1
	6	8	5	1		40	1
5	6	6	5	0		1	1
6	6	6	6	0		1	1
6	6	6	6	0		1	1
		6	6	1	MPEG	17	1
		8		0		10	1

				1	3	1
				0	1	0
				0	2	
5	5	5	5	0	1	1
				0	1	0
5	5	5	5	1	1	
				0	2	1
				0	3	1

Q163 How did you hear about immediat	Q164 Consider taking up ART therapy	Q164Rec Consider taking up ART therapy	Q165 If tested and found to be HIV infected	Q165a If you won't start Anti-retroviral	Q166 Recommend your partner to start	Q166a If you would not recommend your	Q167 What do you think of taking ARVs	Q168 If you were on ARV would you
4	1	0.00	1		1		How to sup	1
4	1	0.00	1		1			1
4	1	0.00	1		1			1
4	1	0.00	1		1			
4	1	0.00	1		1			1
4	1	0.00	1		1		Boost the ir	1
			1		1		Boost the ir	1
3	1	0.00	1		1			1
4	1	0.00	1		1		Right thing	1
2	1	0.00	1		1			1
1	1	0.00	1		1			1
3	1	0.00	1		1			1
1	1	0.00	1		1		Yes	1
1	1	0.00	1		1			1
3	1	0.00	0		1			
3	1	0.00	1		1			1
1	1	0.00	1		1			1
6	0	1.00	0		0		Safe	1
3	0	1.00	1		1			0
2	1	0.00	1		1			1
	1	0.00	1		1		Boost immu	1
1	1	0.00	1				Boost immu	1
								1
1	0	1.00	1		1			1
1	1	0.00	1		1			1
2	0	1.00	1		1		Boosting im	1
	1	0.00	1		1			1
1	1	0.00	1		1		Routine	1
6	1	0.00	1		1			0
1	1	0.00	1		1		Dont know	0
1	1	0.00	1		1			0
6	1	0.00	1		1			1
	1	0.00	1		1	No		0
4	1	0.00	1		1		Boost the ir	1
3	1	0.00	1		1	NA	NA	1
1	1	0.00	1		1		Boost the ir	1

1	1	0.00	1	1		Yes	1
1	1	0.00	1	1			1
15	1	0.00	1	1			1
3	1	0.00	1	1			1
4	1	0.00	1	1			1
1	1	0.00	1	1			1
1	0	1.00	1	1			1
			1	1		Boost the ir	1
	1	0.00	1	1			1
2	1	0.00	1	1		Boost the ir	1
1	1	0.00	1	1		Boost the ir	1
4	1	0.00	1	1			1
4	1	0.00	1	1			1
7	1	0.00	1	1			1
	1	0.00	1	1		Wrong	1
4	1	0.00	1	1		Boost the ir	1
1	1	0.00	1	1			0
4	1	0.00	1	1	Good		1
1	1	0.00	1	1		To attain 9(	1
4	1	0.00	1	1		Boost the ir	1
2	1	0.00	1	1			1
	1	0.00	1	1		Boost the ir	1
	1	0.00	0	1			0
4	1	0.00	1	1		Boost the ir	1
5	1	0.00	1	1		Boost the ir	1
	1	0.00	1	0	Stigma	Boosting in	1
	1	0.00	1	1		Depends wi	1
5	1	0.00	1	1			1
2	1	0.00	1	0			1
4	1	0.00	1	1		Effective in	1
1	1	0.00	0	1			1
3	0	1.00	1	1			0
4	1	0.00	1	1		Boosting in	1
	0	1.00		1			1
1	1	0.00	1	1	Good		1
3	1	0.00	1	1		Yes	1
1	1	0.00	1	1			1
4	1	0.00	1	1			1
	1	0.00	1	1			0
1	1	0.00	1	1		6 Months	1
4	1	0.00	1	1			1
4	1	0.00	1	1		Boost the ir	1
4	1	0.00	1	1			1
	1	0.00	1	1			0
	1	0.00	1	1			0
4	1	0.00	1	1			1
4	1	0.00	1	1		Boost the ir	1
	1	0.00	1	1			1
	1	0.00	1	1			1
4	1	0.00	1	1		Boost the ir	1
4	1	0.00	1	1			1
	1	0.00	1	1			1

4	1	0.00	1		1			1
2	1	0.00	1		1			1
	1	0.00	1		1			0
4	1	0.00	1		1			1
4	1	0.00	1		1			1
			0	Stigma	0	Trust		0
4	1	0.00	1		1			1
1	1	0.00	1		1			1
4	1	0.00	1		1			1
1	1	0.00	1		1	Poor adherence		1
	1	0.00	1		1			0
4	1	0.00	1		1			1
4	1	0.00	1		1		Right	1
	1	0.00	1		1			1
2	0	1.00	1		1			1
	1	0.00	1		1			
4	1	0.00	1		1			1
10	1	0.00	1		1		My health i	1
4	1	0.00	1		1			1
4	1	0.00	1		1			1
3	1	0.00	0		1		Never	0
	1	0.00	1		1		Safe	1
4	1	0.00	1		1			1
1	1	0.00	0		1			1
	1	0.00	1		1			0
1	1	0.00	1		1		Boost immu	1
1	1	0.00	1		1			1
	1	0.00	1		1			0
1	1	0.00	1		1			1
4								
1	1	0.00	1		1	Boosts immunity		1
4	1	0.00	1		1			1
	1	0.00	1		1		Yes	1
3	1	0.00	1		1			1
4	1	0.00	1		1	To boost immunity		1
4	0	1.00	1		1			1
			1		1			1
4	1	0.00	1		1			1
1	1	0.00	1		1		Weaken the	1
1	1	0.00	1		1			1
	1	0.00	1		1			1
2	1	0.00	1		1		Okey	1
	1	0.00	1		1		Wise	1
3	0	1.00	0		0			1
	0	1.00	1		0			0
4	1	0.00	1		1		Prevent the	1
3	1	0.00	1		1			1

			1		1			1
1	0	1.00	1		1			1
3	0	1.00	1		0			0
5			1		1			0
1	1	0.00	1		1		Boost immu	1
1	1	0.00	1		1			1
1			1		1			1
					1			0
1	1	0.00	1		1			
1	1	0.00	1		1			0
	1	0.00	1		1	Own sake		1
	0	1.00	1		1		Effective	1
1	0	1.00	0		0			0
	0	1.00	1		1			1
1	0	1.00	1		1			1
1	1	0.00	1		1		Yes	1
3	1	0.00	1		0			1
5	0	1.00	0	If in good h	0	If in good health No		0
1	0	1.00	0		1			1
	1	0.00	1		1			1
1	1	0.00	1		1		Good healt	1
			0					1
4	1	0.00	1		1			1
1	1	0.00	1		1			1
	0	1.00	0		0			0
1	1	0.00	1		1		Daily prote	1
4	1	0.00	1		1		Nice and go	1
	0	1.00	1		1	Boost immunity		1
2	1	0.00	1		1			1
5	1	0.00	1		1	Good healt	Treatment	1
4	1	0.00	1		1	Good		1
	1	0.00	1		1			1
	1	0.00	1		1			1
4	1	0.00	1		1		Boosts imm	1
	1	0.00	1		1		To maintai	1
1	1	0.00	1		1			1
11	1	0.00	1		1			1
4	1	0.00	1		1			1

	1	0.00	1	1	To maintai	1
3	1	0.00	0	0		0
	1	0.00	1			1
4	1	0.00	1	1		1
1	1	0.00	1	1		1
4	1	0.00	1	1		1
4	0	1.00	1	1		0
			1	1		1
3	1	0.00	1	1		1
3	1	0.00	1	1	Taking ARVs boosts imn	1
1	1	0.00	1	1		1
						1
				1		1
3			1	1		1
3	1	0.00	1	1		1
3	1	0.00	0	1		1
			1	1		1
4	1	0.00	1	1		1
4	0	1.00	1	1		1
3	1	0.00	1	1		1
5	1	0.00	1	1		1
4	1	0.00	1	1		1
1	1	0.00	1	1	Not bad	1
3	1	0.00	0	0		1
4	1	0.00	1	1		1
6	1	0.00	1	1	Boosts imm	1
4	1	0.00	1	1	ARV Boosts	1
4	0	1.00	1	1		1
1	1	0.00	1	1		1
16	1	0.00	1	1		1
1			1	1		
4	1	0.00	1	1	Its ok	1
4	1	0.00	0		Boosts imm	1
4	1	0.00	1	1		1
4	1	0.00	1	1		1
1	1	0.00	0	1		1
1	1	0.00	1	1		1
3	1	0.00	1	1	Boosts imm	1
1	1	0.00	1	1	yes	1
	1	0.00	1	1	yes	1
2	1	0.00	1	1	Suppresion	1
6	1	0.00	1	1	Yes	1
8	1	0.00	1	1	N/A	1
	0	1.00	0	0		1
1	0	1.00	1	1	Boost immu	0

4	1	0.00	1	1	Boosting im	1
4	1	0.00	1	1	Boosting im	1
6	1	0.00	1	1	Healthy an	1
2	1	0.00	1	1	Boosting im	1
2	1	0.00	1	1		1
1	1	0.00	1	1		1
				1		1
1	1	0.00	1	1		1
2	1	0.00	1	1	Boost the i	1
4	1	0.00	1	1	Boost the i	1
	1	0.00	1	1	Bad	1
	0	1.00	1	1		1
8	1	0.00	1	1	Boost the i	1
4	1	0.00	1	1	Reduce risk	1
15	1	0.00	1	1		1
2	1	0.00	1	1		0
4	1	0.00	1	1	Boosting im	1
4	1	0.00	1	1		1
4	1	0.00	1	1	Boost the i	1
1				1		1
9	1	0.00	1	1		1
3	0	1.00	1	1		1
1	1	0.00	1	1	Boost imm	1
1	1	0.00	1	1		1
1	1	0.00	1	1		1
5	0	1.00	1	0		1
			1	1		0
3	1	0.00	1	1		1
2	0	1.00	1	1		1
1	1	0.00	1	1		1
1	1	0.00	1	1	Good	1
						1
4	1	0.00	1	1	Boost imm	1
3	1	0.00	1	1	Good	1
1	0	1.00	1	1	Maintain H	1
	1	0.00	1	1		1
2						
	1	0.00	1	1		1
2	1	0.00	1	1		1
	1	0.00	1	1	Good to prevent more	1
3	1	0.00	1	0		0
1	1	0.00	1	1	Don't know	1
4	0	1.00	1	1		1
1	0	1.00	0	0		0
	1	0.00	1	1		1

1	1	0.00	1		1		1
	0	1.00	1		1		0
	1	0.00	1		1		1
	0	1.00	1		1		1
							1
5	1	0.00	1		1	Reduced vi	1
4	1	0.00	1		1		1
1	1	0.00	1		1		1
4	1	0.00	1		1		1
3	1	0.00	1		1		1
1	1	0.00	1		1		1
	0	1.00	1		1	Keep health	1
	1	0.00	1		1	Reducing chances of ge	1
1							
							1
1	1	0.00	1		1		1
4	1	0.00	1		1		1
3	1	0.00	1		1		1
3	1	0.00	1		1		1
			1		1		
4	1	0.00	1		1	Stay health	1
1	1	0.00	1		1		1
1	1	0.00	1		1		1
	1	0.00	1		1		1
4	1	0.00	1		1		1
1	1	0.00	1		1	Protective	1
1	1	0.00	1		1		1
1	0	1.00	1		1		1
4	1	0.00	1		1		1
3	1	0.00	1		1		1
1	0	1.00	1		0		
	1	0.00	1		1		1
4	1	0.00	1		1		1
1	1	0.00	1		1	Boost immu	1
1							1
1			1		1		1
4	1	0.00	1		1	Boosts imm	1
4	0	1.00	1		1	Boosts imm	1
1	0	1.00	0		1		1
3			1		1		1
4	1	0.00	1		1	Boosts imm	1
2	1	0.00	0	True	1		1

2	1	0.00	0	1		1
3	1	0.00	0	1		1
3	1	0.00	1	0		0
16	1	0.00	1	1		1
	1	0.00	1	1	Yes	
1	1	0.00	1	1		1
4	1	0.00	1	1		

Q169 Do you think ARVs are effective	Q169a If you think ARVs are not effective	Q170 What would prevent you from	Q170Other Other	Q171 Do you think ARV would affect or	Q171a If ARV would affect or disrupt	Q172 Would you tell your partner	Q172a What would be their reaction	Q173 How often is viral load test done
1		5		0		1		6 Months
1		5		0		1		6 Months
1		3		0		1		
1		1		1		1		
1		2		0		1	Surprised	6 months
1				0		1	Neutral	
1		1		1	string	0	separation	12 months
1		2		0		1		6 Months
1		2		1		0		
1		1		0	Separation	1		6 Months
1		1		0		1		6 Months
1		1		0		1	Bad	6 Months
1		3		1		1		6 Months
1		2		1		0		Don't Know
1		5		0		1		6 Months
1		2		0		1	Discriminat	6 Months
1		8		0		1	Scared	No idea
0		2		0		1		13 Months
1		6	N/A	0		1	Positve	6 Months
1		1		0		0	Shocked	
1		2		0		1	Shocked	
1		3		0		1	Positive	
1		1		1		1		
1		1		0		1		6 Months
1		8		0		1		
1		2		0		1	Positive	3 Months
0		11		0		0		
1		1		1	Stigma	0	Negative	N/A
0		3		1		1		
0		4				1		
1		8		0		1	Negative	
1		1		1	When to ta	0	Stigma	6 Months
1		1		0		1	Negative	6 months
1		1		1		1		NA
1		10		0		1	Surprised	6 months

1	1	1		1	Positive	One week
1	3	0		0		
1	15	1		1		NA
1	3	0		1		Yearly
1	1	1		1		
1	5	1	Prevention	1	Know her health	
0	2	0		0		
1	1	1	Difficult to	1	Disappoint	3 Months
1	1	0		1		
1	1	0		0	Stigma	
1	3	0		1	Positive	3 Months
1	2	0		1		6 months
1	3	1	Stigma	1		
1	7	1		1		
1	1	1	Routine	1	Serious	3 Months
	1	0		0	Stigma and	6 months
	1	1		0		
1	1	1		1	Surprised	
1	1	0		0	Stigma	6 Months
1	2	0		1	Positive	6 months
1	2	1		1		3 months
1	5	0		1	Positive	Not sure
0	3	1	Fear			
1	2	0		1	Positive	6 months
1	1	0		1	Negative	3 Months
1	9	1	Time mana	0	Stigma	6 Months
1	1	0		1		
1	3	0		1		3 Months
1	1	0		1		
1	1	0		1		
1	5	0		1		
1	2	1		1		
1	1	1	Changes life	1	Negative	3 Months
1	1	1	Stigma	1		
1	1	0		1	Good	3 Years
1	1	0		0	Negative	6 Months
1	2	1	Partner dis	1	Positive	
1	5	0		1		
1		1		0		
1	2	0		1	Discrimination	
1	5	0		1		
1	2	0		1	Shocked	6 Months
1	5	0		0		
1		1		0		
1	4	0		1	Suportive	3 Months
1	5	0		0		
1	5	0		1	Won't min	6 Months
1		1		0		
1	15	0		0	Don't know	Don't know
1	2	0		1	Supportive	6 Months
1	5	0		0		
1		1		0		

1	5	0	1		
1	2	0	1	Angry	
1		1	0		
1	9	0	1	Shocked	Don't know
1	5	0	0		
1	8	1	0		
1	5	0	1		
1	1	1	1	Stigma	
1	5	0	1		
1	1	0	1	Positive	3 Months
1		1	0		
1	5	0	1		
1		0	1	Shocked	
1		1	0		
1	2	0	0		
1	5	0	1		
1	12	0	0	Negative	6 Months
1	5	0	1		
1	1	0	0	Once	Being sick
1	1	1	0		
1	1	1	1	Sad	
1	5	0	1		
1	1				
1		1	0		
1	2	0	1	Happy	6 Months
1	1	0	1		
1		1	0		
1	2	0	1		
1	2	1	1	Neutral	Sometimes
0	1	0	1		1 per year
1	1	0	1	shocked bu	3 Months
1	2	1	1		
0	2	0	1		
1	2	0	1		
1		0	0		
1		1	1		
1	3	0	1	Normal	
1	2	0	0		
1	1	0	0		
1	5	0	1	Sad but understanding	
1	5	0	1	Positive	Monthly
1	3	1	1		
1	4	0	1		
1		0	1	Good	
1	3	1	1		

0			1		1		
1		4	0		0		
0		3	0		1		
1		3			0		
1		1	0		1		Don't know
1		14	0		1	Would be ok	
1		4	0		1		3 Months
1		3	1		1		
1		5	0		1		
0	Its 99.9% su	5	0		1	Supporful	LDL
1		2	0		1	Nothing	
0		3	0		0		
1		2	0		1	Nothing	
1		1	0		1		
1			0		1		6 Months
1		3	1		1		6 Months
0		8	0		0		
1		5	0		1		
1		3	0		1	Stigma	
1		1	1	Side effects	0	Stigma	1 Year
		3					
1		4	1		1		
1		2	0		1		
0			0		0		None
1		4	0		1	Don't know	None
1		1	0		1	Happy	
1		4	1		1		Don't know
1		3	0		1		
1		3	1		1		1 Month
1		3	0		1		6 Months
1		1	0		1	Positive	1 Year
1			0		0		2 Months
1		1	0		1	Shock	6 Months
0		3	0		0		3 - 6 Month
1			0		1		6 Months
1		3	0		1		I don't know
1		5	0		1		6 Months

1	1		1	When on AI	1	Break up wi	Not sure
1	4		1		1		
1	6	Nothing	1	Change sexu	1		6 Motnhs
1	4		0		1	Guidance a	Not sure
1	1		0		1	Good	Don't know
1	1		1	Stigma	1		
0	5		0		0		
					1		
1			0		1		3 Months
1	1		0		1	Stigma	
1	1		0		1		Low
1	5		0		1		
1	4		0		1		
1	5		0		1		
1	3		1		1		
	2		1		0		
	2		0		1		
0	2		0		1		
1	2		0		1		No idea
1	1		0		1	N/A	N/A
1	1		1	Stigma	0		N/A
1	5		0		1		
1	5		0		1	Cool	
0	1		0		1		
1			0		1		
1	5		0		1	Shock and f	6 Months
1	5		0		1	Schocked a	6 Months
1	7		0		0	Deget	6 Months
1	5		0		1		6 Months
1	16		0		1		
1	4		0		0		Monthly
1			0		0		
1	1		0		1	Positive	1 Year
1	1		0		1	Shocked	
	1		0		1		
1	1		0		1		
1	1		1	Stigma	0		6 Months
1	1		0		1	Serious	6 months
1	1		0		1	Bad	No idea
1	3		0		1	impassive	No idea
1	3		0		1	Positive	6 Months
1	8		0		0		6 Months
0	1		0		1	No	
1	4		0		1		

1		1		0		1	Positive	3 Months
1		3		1		0	Shocked	6 Months
1		10		0		0		3 Months
1		1		0		0	Negative	
1		4		1		1		
1		3		0		0	Fear	Weak
1		2		0		1		
1		1		0		1		
1		3		0		1	Shocked bu	6 months
1	Reduce Vira	1		1	Reduce Vira	1	Disappointed	
1		15		1		1		3 Months
1		3		0		1	Don't know	3 Months
1		2		0		1	Stress	6 months
1		5		0		1	Shocked	
1		3		0		1	Positive	6 months
0		4		1		1		
1		3		1		1		6 Months
1		1		1	Fear	0	No disclosu	Negative
1		3	Discuss wit	1	Drug burde	1	Negative	Monthly
0		2		1		1		
1		11		1		1	Positive	6 Months
1		1		1	Will have tc	1	Shocked	
1		1		1	Stigma	1	Hostile	
1		3		1		1		
1		3		1	Don't know	1	Positive	3 Months
1		2		1	Have to tak	1	Positive	Not aware
0				0		1		
1		3		0		1	Not sure	Forgetting
1		5		0		1	Not sure	Very often
1		1		0		1		6 Months
1		2		0		1	Fear	3 Months
1		1		0		1		
1		1		0		0		No idea
0	not heard	2		0		1	Fear	3 Months
0		1		0		1		
1		13		0		1	Don't know	Not sure
1		3		0		0		
1		4		0		1		No idea
1		3		1	Body horm	1	Shoked	Not sure
0		11		1		1		None
1		3		1	Fear	0	Don't know	
1		2		0		1		
0		3		0		0		
		2		0		1		

1		1	0		1		Donn't kno
1		1	1	Daily duty (	1	Shocked	
1		1	0		1	Positive	Year
1		1	1		1		6 Months
1		12	1	Hate medic	1	Dead	
1		5	0		1	Don't know	3 months
1		1	0		1		6 Months
1		1	1		0		
1		1	0		0		3 Months
1		2	1		1		
1		4	0		0		3 Months
1		1	0		1	Doubt	Yes
1		5	0		0	Badly	
1		1	1		0		
1		5	0		1	Accept and enroll for m	
1		5	0		1	Positive	Not sure
1		3	1	Not easy to	1	Suprised	
1		3	0		1	Shocked	
1							Not aware
1		3	1	Self stigma	0	Regreats &	No informa
1		1	0		1		
1		3	0		0		
1		8	0		1	Scared	Not applica
1		3	1		1		
1		5	0		0	Fear	
1		1	0		1		
0		3	1		0		
0		2	0		1		
1		1	0		0		
1		1	1		1	Support	
1		1	1	Suspicion	1	N/A	Routinely
1		4	0		0	Spread rou	3 Months
1							
1		2	0		1	Okey	1 Year
1		1	0		1	Supportive	1 Year
1		2	0		0	Excommun	N/A
1		1	0		0		
1		1	0		0	Run away	
1		3	0		1		
0	Stigma	1	1		0	Yes	No

0	2	1	1		
1	1	0	0		
1	1	0	0		
1	17	0	1	N/A	
1	1	0			
1	3	0	1	Shoked	3 Months

Q174	Q175	Q176	Q176a	Q176b	Q176c	Q176d	Q176dIf	Q177
What would prevent you from	What fear would you be having on	Would you be knowing your viral	If you know your viral load results	If on ARVs on average how many days do	If you miss what are the common	Have you ever experienc ed stigma	If ever experienc ed stigma in the	If you were on ART would you prefer
None	Nothing	1	1	None	Viral load r	0		1
		1	1	None	Nothing	0		1
		0				1	Medical fac	1
						0		0
		1				0		1
Use of drug	Side effects	1	1	Two	alcohol	0		1
						0		1
								1
place to sta	side effects	1	1		loss of weig	1	friends	1
Drug and su	Side effects	1	1					
		1	1			1		0
Taking it of	Fear	1	1	None	Work	1	community	0
None	None	0				0		0
Stigma	Discriminat	1	2					1
Stigma	Discriminat	1	2					1
'		0						0
N/A	N/A							1
		1	1			1	Friends	
Lack of foo	Discriminat	1						0
Nothing	None	0	2	No	Can not	0		0
N/A	N/A	1	1	1 Day	Lateness	0		1
Access	Stigma							1
Transport	Stigma							1
Being negat	Diet	0				0		
		1	1			1		1
Work	People	0						1
		0						
Reminders	Neglection	1	2					1
		0	2			0		0
Transport	Enrollemen	0				0		1
		0	2			0		0
		1	1			0		0
Forgetting		0				0		1
Stigma	Stigma	0		None	Lateness			
Poor adher	Stigma	0						1
NA	NA	1	2	NA	NA	0		0
Job	Stigma	1	1	One	Late from w	0		1

	Side effects	1	1	One	Death	1	No	1
Finance		1	1	One		0		1
NA	NA						NA	
Fear		0				0		1
		1	1			1		1
		1	1			1		1
		1				0		0
Forgetting	Side effects	0		NA	NA	0		0
								1
						1		
Lack of nut	Stigma	1	1					1
Alcohol		1	1		None	0	Not applica	1
		0				0		0
		1	1			1		1
Time	Side effects	1	1			0		1
Envirnmen	Daily pill bu	1	1	N/A	N/A		N/A	
		1	2			1		1
Alcohol		1						
Time	Stigma	0		None	Lack suppre	1	Friends	1
Lack of kno	None	0				0		
		1	1			1		1
PrEP	Side effects	1						1
Not willing		0				0		0
Knowledge	Stigma	0			Not at all	0		1
Stigma	Side effects	0				0		1
Time mana	Diet	0					Partner	1
Alcohol		1	2			1	Friends	1
Side effects	Disclosure			None	N/A	0		1
		1	1	Yes	Nothing	0		0
Stigma								1
		0	2			0		1
		0	1			0		1
Fatigue	Lack of kno	1	1			0		1
Poor adher	Discriminat	0						
Stigma	Stigma	1	1	Last suppression		1	Friends	1
Time	Stigma	1	1	1 Day	Travelling	0		0
Inavailabili	Side effects	0				0		1
		1	1			0		1
								0
		0						1
		1	1			0		1
	Side effects	1	1	1 - 2 times	Forgot	1	Peers	1
		1	1			0		1
								0
Work time	N/A	1		N/A	N/A			1
		1	1			0		1
If negative	Drug burde	1	1	None	May be dru	0		1
								0
	Stigma	0						1
Adherence,	Side effects	1	1	2 days	Alcohol anc	1	Friend	1
		1	1			0		1
								0

		1	1			0		1
Stigma		0				0		1
								0
Alcohol	None	1	1	None	None	0		1
		1	1			0		1
								0
		1	1			0		1
		1	1			0		1
Poor adher	Stigma	0				0		1
								0
		1	1			0		1
	None	0				0		1
								0
		1	1			0		1
Stigma	Stigma and	0		3 Daya	adhering w	1	Friends	1
		1	1			0		1
		1	2	None	Drunk	0		1
	No	1	2		Poor adher	0		1
						1	Health care	1
		1	1			0		1
								1
								0
Fear	Its a life tim	1	1	No skipping		0		1
								0
		1	2			1		0
						1		1
Nothing		1	1					
Nothing	Side effects	1	1	Not missed	Never miss	0		1
Yes	Side effects	1	1	Never		1		1
		1				1		1
			1			1		1
		0				0		1
		0	2					
		1				0		1
	Feeling a sh	0				0		1
Fear	Shame	0				0		1
		1						1
Work	Stigma	1	1					
Nothing	No fear	1	2					1
		1	2			0		0
		0	2			0		1
		1	1					

		1				1		1
						0		0
		0	2			0		0
						0		1
None	None	1	1			1		1
Stigma	Afraid of kn	1	1			0		
		1		Everyday		0		0
		1	2			0		
		0				0		0
		1	1			0		0
						1		1
		0		Daily				
Nothing	None	0				0		1
		0	2			0		1
Nothing	Nothing	0				0		0
		1	1			1		1
N/A		1	2					1
Stigma	Discriminat	1	1			1	Friends anc	0
Finance		0						
Boredome	Stigma	0				0		1
Stigma	Stigma	1	1					0
		1	1			1		1
		1	1			0		1
	None	0		None	None	0		1
None	None	1	1	Don't know	No	0		0
Nothing	No fear if it	0		Use ARV		0		1
Time and a	Side effects	0						1
		1	1			1		1
Nothing	No	0		None		0		1
Alcohol		1	1	None	None	1	From societ	1
Stigma	Lack of kno	0	2			0		1
	Side effects	1	1	1 - 2 Days	Forgetting	0		1
Fear	Stigma	1		N/A	N/A	0		0
Fear	People will	1	1	1 Week	Forgetting	0		1
		0				0		1
Stigma	Side effects	1	1	None	No	1	Colleagues	
None	None	1	1			0		1

Forgetfulne	Stigma	0		No idea	N/a	0		1
		0				1		0
Nothing	Side effects	0				0		1
None	No fear	1				0		1
'	good	1	1	Don't know	Can be sick	1		1
		1	2		Dizzness	1		1
Cost		1	2		Cost	0		0
None		0		2 Months	None	0		1
Money	Discriminat	0				1	My friends	1
Healthy bo	Nothing	1	2	3 Months	No	1		0
								1
								0
		0				0		
						1		1
		1						
		1	2			1		0
Discriminat	Fear	0		No idea	Lack	0		1
N/A	N/A	0		N/A	N/A	0		0
N/A	N/A	1	1	N/A	N/A		N/A	
		1				0		1
Side effects	Stigma	1	1			0		1
		0	2			0		0
								1
N/A	N/A	1	1	N/A	N/A	1	Partner	1
Time	Side effects	1	1	N/A	N/A	1	Partner	1
Time and Jc	Its burden	1	1	None	Reduce immunit	0		1
Nothing	Side effects	1						1
		1						
						0		1
Discriminat	Drug burde	1	1	1 Month	None	0		1
		1	1	None		1	Community	
Time	Side effects							1
	Stigma	0						
								0
None	None	1	2			0		1
Stigma	Stigma	1	1	No day		0		1
Use alcohol		1	2	several		0		0
Drug abuse	Side effects	0		no idea	N/A	0		1
Job	people	0			working ho	0		1
Drugs/Diet	Length of m	1	1	None				
Transport	No fear	1	2	No	N/A	0		0
		0	2			0		1
Lack of kno	Stress	0		1 Day	Weakness	0		0

Access	Side effects	0						1
Alcohol	Confidentia	1	1	1 Day	Alcoholism	0		0
Stigma	Side effects					1	Friends	1
Stigma and	Side effects					1	Partner	1
		1	1			0		1
ARV	None	1	1			0		1
								0
		0	1			0		0
Fear	Side effects	0				0		1
	Side effects	1	1	Once	Stress	1		1
People arou	Side effects	0	1			0		0
PEP	People arou	0						
Nutrition	Stigma	1	1	Once	Missing foo	1	Communit	1
	Side effects	0				0		1
	Side effects	1	1	NA	Ignorance	0		1
		1	2			0		0
Stigma and	Side effects	1	2			1	Peers	1
Forgetting	Drug burde	0						0
Travelling a	Left by part	1	1	1 -2 days a r	travelled	0		1
		1				1		1
Stigma and	Side effects	1	1	None		0		1
Forget		1	1	None	Forgetfulness			0
Drugs	Stigma	0				0		1
			1			1	Teacher	1
		0	1	4 days		1	Teacher	1
Busy sched	Side effects	0		Not aware	Not aware	0		0
		0				0		
		0						1
Nothing	Side effects	1	2					0
Privacy	Side effects	0						1
Allergic to doctors advi		1	2	None		1	Friend	1
Under drug influence								
Allergy	My partner	1	2	None		1	Friend	1
		1				0		0
Side effects	Side effects	0		N/A	N/A	0		1
Fear & stigma		1	1			0		1
Nothing	Lack appeti	1	1	No		0		1
Side effects	Body horm	0		N/A	N/A	0		1
None		1	1			0		1
		1	1					1
						0		1
Lack of kno	Side effects	1						
		0	2			0		0
		0						0

Nothing		1	1			0		1
Stigma and	Cost	0						1
Effects	Stigma	0				0		1
Time	Stigma	1	1		Time	0		
Not availab	Lack of kno	0						
Time	Side effects	0						
Fear		1	1			0		1
Fear		1	1			0		1
								1
No	No	1	1	No	No	0		
Yes	Side effects	1	1	Never	Should not	0		
	Side effects	0						
						0		
		0						
Nothing	Side effects	0						
None	No fear	1				0		1
Side effects	None	0						1
		0						
Stress	Tension	0		No information		0		1
Once	Discriminat	1	1	None		1		1
		0				0		1
Stigma	Drug burde	0						1
								1
		1	1			1		1
Nothing	No	1	1	No day		0		1
		1	1			0		1
		1	1			0		1
		1	2			1		0
						0		0
		1	1			1	Friends	1
						0		1
		0						
Forgetting	Doubt	0				0		1
Stock outs	Peer pressu	1	1		time	0		1
							N/A	0
Alcohol	Side effects							1
Work	Side effects							1
N/A	Side effects	1	1	2 Months	Forgetting	0		1
		0				1	Friends	
		1	1			0		0
	Start previr	1	2	Week days		0		1

		0		1		0
		1	2	0		1
		1	2	0		1
Money con	Being doub	1	1	1	N/A	
Access	Stigma			0		1
				0		1
Lack of knowledge		1	2	0		1

Q178	Q179
Do you have any additional question	DO you know any other method of
No	No
No	No
	Condoms
Has there b	Condom and lubricants
If self test w	Absteinance
No	
No	Faithful
No	
No	No
No	None
No	No
	Avoid sharing sharps
	Absteinance
No	No
None	No
N/A	
No	No
No	No
No	Absteinance
No	Absteinance

No	
No	No
	NA
	Condom use
	Condom use
NA	NA
No	No
No	Abstainance
	No
	Condom use
No	No
	Condom and lubricants
	Faithfulness
No	Abstainance
	No
None	PrEP
No	Absteinance
N0	No
No	No
	No
No	No
No	No
	No
No	
None	
N/A	Abstainance
No	Being faithful
No	
Want more	Condom and lubricants

	Abstainance
None	Abstainance
No	Abstainance
No	Abstainance
No	No
No	No
No	Abstainance
No	No
	Abstainance
No	No
	Negative
No	No
	Use of condom
	Abstainance
No	No
No	No
	No
No	
No	Avoid sharing
No	No

No	No
No	No
None	No
	None
	PrEP
	Abstainance
No	
No	No
No	Abstainance
No	No
No	Abstainance
No	Condoms
No	Abstainance
No	No
No	No
No	No
No	
No	No
None	None
	Feeding unwell
No	No
	Condom
None	Protective

No	Abstainance
None	None
No	No
	Abstainance
No	No
No	
No	Condom use
No	No
None	None
	Using condom
No	
N/A	N/A
	N/A
No	
N/A	No
Early virus [illegible]	ABC
All ARV tak[illegible]	N/A
No	No
No	No
No	
	Abstainance
No	
No	
N/A	Absteinance
No	No
	PrEP

	Absteinance
No	No
No	
No	No
No	No
	None
Can t	Condom use
	Condom use
No	No
No	No
No	
Will I conti	None
	Condom and Lubricants
No	Absteinance
	No
No	Abstainance
No	No
No	Abstainance
Need to take care of the unemployed	
No	No
	Abstainance
N/A	N/A
No	No
No	
	Abstainance and faithfulness to one partner
No	
N/A	Abstainance
No	No
N/A	Abstinance
No	
No	No
	Smoke weed
No	No

No	No
No	No
No	

What is viral load and suppressed and unsupressed

No	Abstainance
No	No
	PrEP
No	No
None	
No	
	Abstainance
No	Sefl Control
No	No
No	Abstainance
No	No
None	
None	
N/A	N/A
No	No
N/A	N/A
No	No
No	Abstainance
N/A	N/A
No	No
No	Prevention

No	No
No	No
